# In Situ Formation of an In–Zn Interface Layer Enables Aqueous Zinc‐Ions Batteries with High Capacity Retention

**DOI:** 10.1002/advs.74972

**Published:** 2026-04-14

**Authors:** Youwei Jiang, Jinghao Li, Jie Huang, Tan Qiu, Xiuwen Song, Fujun Deng, Yongxue Wang, Yue Ming, Ziping Wu, Qinyou An, Yaoguang Rong

**Affiliations:** ^1^ School of Chemistry Chemical Engineering and Life Sciences Wuhan University of Technology Wuhan Hubei China; ^2^ State Key Laboratory of Advanced Technology For Materials Synthesis and Processing Wuhan University of Technology Wuhan Hubei China; ^3^ School of Integrated Circuits Shenzhen Polytechnic University Shenzhen Guangdong China; ^4^ College of Rare Earths Jiangxi University of Science and Technology Ganzhou China

**Keywords:** aqueous zinc ions battery, high capacity retention, in situ formation, In–Zn interface layer

## Abstract

For aqueous zinc ion batteries (AZIBs), inhibiting side reactions is the key to enhance the stability. Particularly, the issues of uneven deposition and hydrogen evolution reaction have attracted intensive attention. We propose to construct an In–Zn interface layer between the separator and anode to induce uniform deposition of metallic zinc. Being different from the traditional direct deposition method, we firstly sputter indium zinc oxide (IZO, In_2_O_3_:ZnO = 9:1 wt.%) that demonstrates superior zincophilicity on the separator surface as an activator. Then, the activator is reduced to indium during cycling and merges with the anode, in situ forming an In–Zn interface layer. The In–Zn interface layer accelerates the transport of Zn^2+^, facilitates the nucleation/growth of zinc. Meanwhile, the potential of hydrogen release is reduced from −0.051 to −0.077 V. For symmetrical cells (Zn(OTf)_2_ electrolyte), a lifespan of over 5000 h at a current density of 1 mA cm^−2^ is achieved. For V_6_O_13_‐Zn full cells, a capacity of 311.77 mAh g^−1^ at 2 A g^−1^ is obtained, and the capacity retention reaches 80.72% after 6000 cycles (413.17 mAh g^−1^ at 0.1 A g^−1^, 82.24% after 633 cycles). Notably, this strategy is universal and also works for AZIBs using ZnSO_4_ electrolyte and other cathodes.

## Graphical abstract

This work demonstrates an indium zinc oxide (IZO)‐modified separator applied to aqueous zinc ion batteries (AZIBs), which in situ generates an In–Zn interface layer to stabilize the separator/anode region during the cycling process. The In–Zn interface layer provides richer nucleation sites for Zn^2+^ and suppresses side reactions. The assembled AZIBs achieved a capacity retention of 80.72 % after 6000 cycles at 2 A g^−1^.

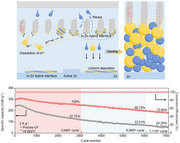

## 1 Introduction

Rechargeable zinc‐ion batteries have garnered extensive attention in the field of electrochemical energy storage due to their high theoretical capacity and abundant zinc resources [1, 2, 3, 4, 5, 6, 7, 8], among which aqueous zinc ion batteries (AZIBs) are considered as a highly promising candidate for next‐generation large‐scale energy storage systems, owing to their inherent safety, environmental friendliness, and cost‐effectiveness compared to conventional lithium‐ion batteries [9, 10]. The use of aqueous electrolytes eliminates the risks associated with organic solvents, making AZIBs particularly suitable for grid‐scale applications where safety and scalability are paramount [11]. Additionally, metallic Zn anodes offer a high theoretical capacity (820 mAh g^−1^ and 5855 mAh cm^−3^) and a low redox potential (−0.76 V vs. SHE), further enhancing their appeal for high density energy storage solutions [12].

Nevertheless, the practical application of AZIBs still encounters major challenges due to the unstable electrolyte/electrode interface layer. Particularly, uncontrolled Zn^2+^ deposition and the hydrogen evolution reaction (HER) at the Zn anode have attracted intensive attention [13]. The interactions precipitate the formation of by‐products (such as Zn_x_OTf_y_(OH)_2x‐y_·nH_2_O) [14] and promote heterogeneous Zn deposition [15], thereby leading to sluggish kinetics and accelerating dendritic growth. Consequently, the irreversible interfacial reactions result in low Coulombic efficiency (CE) and deteriorate the system of batteries during the plating/stripping of Zn^2+^, impeding the further progress of AZIBs in practical applications [16, 17].

To address these issues, various strategies have been developed, which mainly focus on cathode design [18, 19], anode modification [20, 21, 22], and electrolyte optimization [23, 24, 25, 26]. Liu et al. reported an amorphous organic‐hybrid vanadium oxide, featuring one‐dimensional chains arranged in a disordered structure with atomic/molecular‐level pores for promoting hierarchical ion diffusion pathways and reducing Zn^2+^ interactions with the solid skeleton [27]. Notably, coating protection layers on the Zn anode and homogenizing the electric field at the electrolyte/anode interface have proven effective in enhancing the cycling stability of AZIBs [28, 29]. Besides, polyimide covalent organic frameworks [30] and metallic layers (e.g., In, Cu, Ti) [31, 32, 33] have also been demonstrated to control the nucleation processes, electric fields, and ionic flux on the electrode surface, thereby improving the performance and stability. Electrolyte engineering is an effective method for optimizing the interface of AZIBs. Pastel et al. designed two functionalized co‐cations that offer a partially fluorinated pyrrolidinium cation that strongly suppresses parasitic reactions such as HER and forms tortuous deposits, while an ether‐functionalized ammonium cation supports more uniform stripping and plating, thereby limiting dendrite formation [34]. Also, treating the surface of separator has shown similar beneficial effects. Huang et al. reported a cellulose nanofibers‐ZrO_2_ separator that could provide a directional electric field and thus induce homogeneous deposition of Zn metal [35]. These works highlight the essential role of surface and interface modifications for enhancing the performance of AZIBs.

Herein, we attempt to develop a strategy that is able to simultaneously modify the interfaces of separator/electrolyte and electrolyte/Zn anode. Firstly, indium zinc oxide (IZO, In_2_O_3_:ZnO = 9:1 wt.%) is deposited on the surface of a glass fiber (GF) separator via magnetron sputtering as an activator. The IZO layer is highly conductive and zincophilic. During cycling, the IZO activator emerges with the Zn anode and in situ forms an In–Zn interface layer. The conductive In–Zn interface layer with high zincophilicity could help to homogenize the electric field (as simulated by the COMSOL Multiphysics simulations). Also, the interface layer significantly improves zinc deposition kinetics (nucleation overpotential of 33.0 mV, growth overpotential of 26.6 mV), effectively suppresses HER (potential reduced from −0.051 to −0.077 V), and induces zinc metal deposition within the interface layer. In our previous works, we have devoted great efforts to the optimization of vanadium oxide cathodes for high‐performance AZIBs [23, 36]. The Zn(OTf)_2_ electrolyte has always been one of the most suitable electrolytes for vanadium oxide cathodes, effectively suppressing the dissolution of vanadium [37, 38]. When the interface layer is employed in V_6_O_13_‐Zn full cells, a capacity of 413.17 mAh g^−1^ is obtained at 0.1 A g^−1^, and the capacity retention reaches 82.24% after 633 cycles. At 2 A g^−1^, a capacity of 311.77 mAh g^−1^ is obtained, and the capacity retention reaches 80.72% after 6000 cycles. We also matched other typical systems in AZIBs to verify the effect of the In–Zn interface layer, including cathodes such as V_2_O_5_, NH_4_V_4_O_10_, and PANI, as well as the common electrolyte of ZnSO_4_, and achieved significant performance improvements.

## 2 Results and Discussion

### 2.1 In Situ Formation of the In–Zn Hybrid Interface Layer

To suppress side reactions and enhance the cycling stability of AZIBs, we attempt to introduce a protective interface between the separator and Zn anode. Previously, various metal coating strategies for Zn anodes have been reported [39, 40]. Particularly, indium has low resistivity and high hydrogen evolution overpotential (−0.342 V vs. SHE) [41, 42] and has been prepared InZn alloys to inhibit the corrosion of Zn metal [43]. Also, indium oxides (In_2_O_3_) and their derivatives have demonstrated promising potential as a protective layer for zinc anode due to their high stability in aqueous environments and high zincophilicity [44, 45, 46]. Notably, the highly conductive indium tin oxide (ITO) facilitates a uniform in‐plane electric field and thus promotes uniform migration of zinc ions [47]. Similarly, IZO uniquely integrates the core strengths of In_2_O_3,_ including high stability, zincophilicity, and anti‐corrosion capacity, as well as the advantage of ZnO, which is native compatibility with the Zn anode. This unique combination of attributes establishes IZO as a promising candidate for Zn anode interface modification in high performance AZIBs.

The nucleation overpotential serves as a pivotal parameter for quantifying the zincophilicity of an electrode. Regulation of this parameter enables effective suppression of zinc dendrite growth, thereby enhancing the Coulombic efficiency and cycling stability of AZIBs [48]. On the other hand, the growth overpotential primarily characterizes the ion diffusion/mass transfer resistance and interfacial charge transfer kinetic barriers, which exert a direct impact on the uniformity and microtopography of the electrodeposited zinc layer [49]. Given the critical roles of these two overpotentials in determining Zn anode performance, optimizing both through interface modification is essential for high‐stability AZIBs.

Introducing a conductive layer onto the separator or the Zn anode could effectively homogenize the electric field and facilitate the uniform deposition of zinc ions, thereby regulating Zn electrodeposition and suppressing dendrite formation [50, 51]. The selection of the substrate for the protective interface is of crucial importance. Modification of the Zn anode surface may directly act on the deposition site but risks poor interfacial adhesion and hindered volume expansion of Zn during cycling, which could elevate the growth overpotential by restricting ion transport [52]. In contrast, coating on the separator can indirectly modulate the electric field between the anode and separator while avoiding direct contact with the Zn anode, potentially reducing the nucleation overpotential through improved zincophilicity and lowering the growth overpotential via unobstructed ion pathways [53]. To clarify the optimal modification strategy and its impact on the two key overpotentials, we systematically compared both substrates by sputtering pristine In_2_O_3_, ITO (In_2_O_3_: SnO_2_ = 9:1 wt.%) and IZO (In_2_O_3_:ZnO = 9:1 wt.%) onto either the Zn anode to form Zn@In_2_O_3_, Zn@ITO, Zn@IZO or the GF separator to form GF@ In_2_O_3_, GF@ITO, GF@IZO as shown in Figure S1.

**FIGURE 1:**
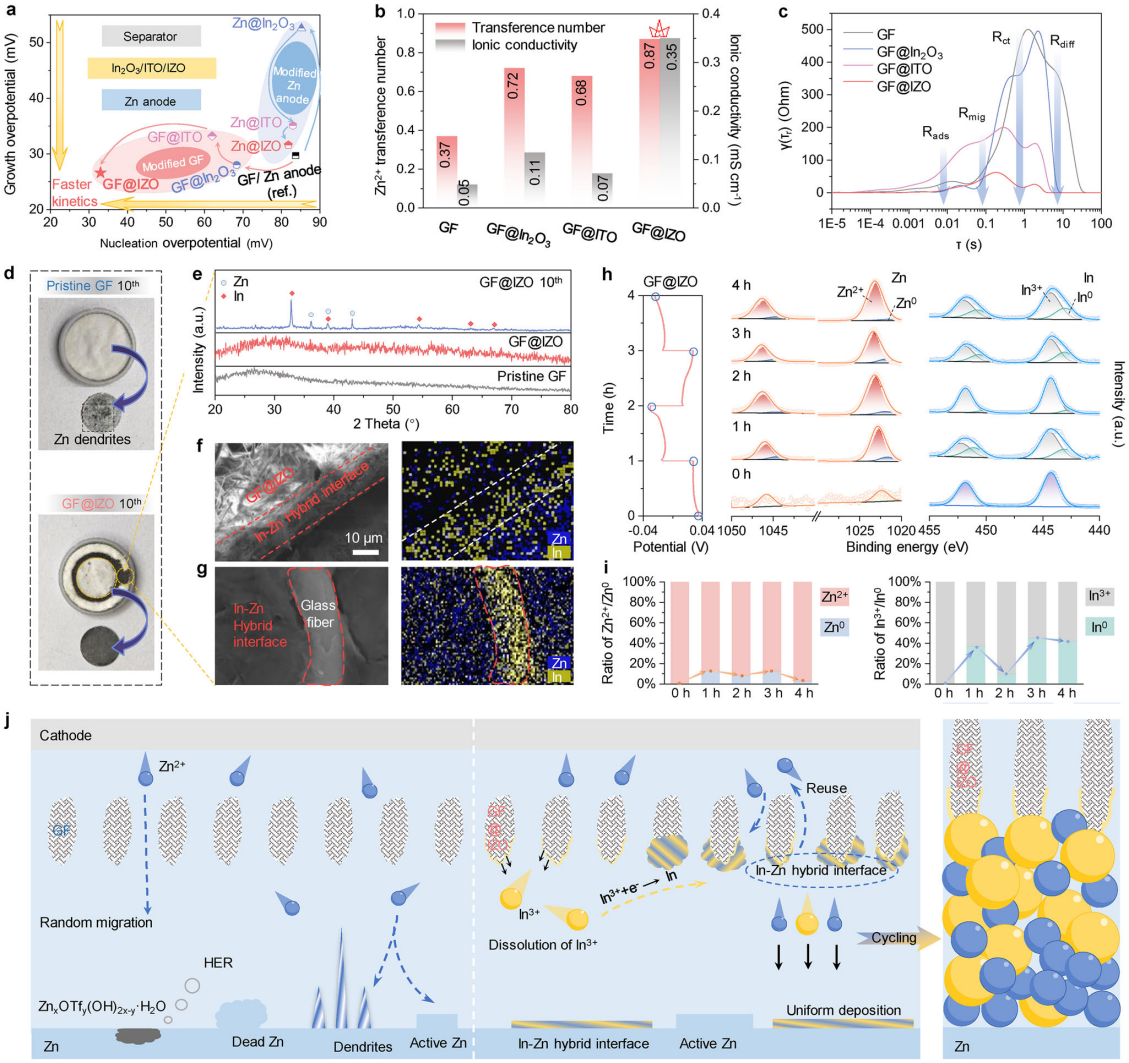
(a) Comparison of nucleation overpotential and growth overpotential of In_2_O_3_ and its derivatives sputtered on Zn anode/GF for the assembly of symmetrical cells. (b) Comparison of Zn^2+^ transference number and ionic conductivity of In_2_O_3_ and their derivatives sputtered on GF. (c) DRT analysis of Zn–Zn symmetrical cells with different separators. (d) Optical images of the separator and Zn anode after 10 cycles. (e) XRD pattern of GF@IZO after cycling, GF@IZO, and pristine GF. (f) Cross‐sectional SEM and (g) details with GF@IZO after cycling. (h) XPS spectra (Zn 2p and In 3d) of the GF@IZO at the initial 2 cycles. (i) The valence state dynamics of In and Zn within the GF@IZO system. (j) Schematic illustrating the function mechanism differences between the separator/anode interface in the full cells with pristine GF and GF@IZO.

Benefiting from the high conductivity of these metal oxides, the modification is expected to accelerate reaction kinetics and reduce charge transfer barriers. The conductivity of In_2_O_3_, ITO, and IZO is compared in Figure S2. We assembled Zn–Zn symmetric cells to evaluate the nucleation overpotential and the growth overpotential as key metrics for deposition behavior, as presented in Figure 1a and Figure S3. The unmodified GF/Zn anode exhibits a nucleation overpotential of 83.7 mV and a growth overpotential of 29.7 mV. This phenomenon is attributed to interfacial mismatches and restricted ion transport that compromise kinetic performance [54].

In contrast, modification on the GF surface induces significant reductions in both nucleation overpotential and growth overpotential for all three materials, facilitating uniform and rapid Zn deposition. This outcome confirms that the separator is a superior modification substrate compared to the Zn anode, as it synergistically optimizes zincophilicity and ion transport kinetics. The reduced nucleation overpotential reflects enhanced zincophilicity, while the lowered growth overpotential indicates improved ion transport kinetics, and these two aspects are the core factors governing Zn anode performance. Among the three materials, GF@IZO exhibits the most remarkable reductions in both overpotentials, further confirming the unique superiority of IZO for constructing high‐performance protective interfaces in AZIBs.

We further characterized the Zn^2+^ transference number ($t_{Zn^{2+}}$) and ionic conductivity of Zn–Zn symmetrical cells integrated with the three modified separators (Figure 1b and Figure S4). These two parameters are core factors governing ion transport behavior in AZIBs [55]. Notably, GF@IZO delivers a Zn^2+^ transference number of 0.87. This value far surpasses that of pristine GF (0.37), GF@ITO (0.68), and GF@ = In_2_O_3_ (0.72). A high Zn^2+^ transference number enables the preferential transport of Zn^2+^ while restricting the migration of other ionic species. This effect suppresses side reactions more effectively [56].

In terms of ionic conductivity, GF@IZO exhibits a value of 0.35 mS cm^−1^. This value is 7, 3.5, and 5 times higher than that of pristine GF (0.05 mS cm^−1^), GF@ITO (0.10 mS cm^−1^), and GF@In_2_O_3_ (0.07 mS cm^−1^), respectively. This enhanced ionic conductivity facilitates efficient Zn^2+^ transport within AZIBs. It reduces internal resistance and improves charge–discharge efficiency [57].

To illustrate the effect of high zincophilicity and Zn^2+^ transfer ability on the internal resistance characteristics of AZIBs, we assembled Zn–Zn symmetrical cells by electrochemical impedance spectroscopy (EIS) tests (Figure S5). For the Nyquist plots, the overall impedance reflected by the curve extent follows the order: GF > GF@In_2_O_3_> GF@ITO > GF@IZO. Then, the distribution of relaxation times (DRT) analysis of Zn deposition was used to decouple the kinetic behavior of Zn^2+^ in different separators (Figure 1c). Analysis of the DRT reveals that the peaks at relaxation times τ of around 10^−2^, 10^−1^, 10^0^, 10^1^ s correspond to the adsorption and dissociation of Zn^2+^ (R_ads_), the migration process of Zn^2+^ on the anode surface (R_mig_), the charge transfer process of Zn^2+^ (R_ct_), and the diffusion process of Zn^2+^ (R_diff_), respectively [58]. These peaks reflect resistance at key steps of Zn^2+^ deposition. Unmodified GF exhibits strong peaks for all resistances, indicating high kinetic barriers across the full deposition process. GF@In_2_O_3_ and GF@ITO reduce resistance in selected steps but show limited improvement. In contrast, GF@IZO displays the weakest peaks for all resistances, demonstrating synchronous optimization of the full Zn^2^+ deposition kinetic chain. The kinetic regulation explains the dual reduction of nucleation/growth overpotentials for GF@IZO.

Post‐cycling characterization further visualizes the regulatory effect of IZO modification on Zn deposition behavior. Figure 1d displays the Zn anode and separator morphologies after 10 cycles. After cycling, the pristine GF showed no obvious changes, whereas numerous Zn dendrites appeared on the surface of Zn anode. Compared to pristine GF, GF@IZO revealed a distinct color transformation in the IZO layer from yellow to black. Furthermore, upon disassembling the cell and separating the Zn anode from the separator, the black substances exhibited partial adhesion, with the discoloration primarily concentrated at the central region of the separator before gradually diffusing outward. And the black substances also emerged on the surface of Zn anode. To confirm the components of the black substances, X‐Ray Diffraction (XRD) analysis revealed the amorphous and crystalline nature of the pristine GF, GF@IZO, and GF@IZO after cycling. The XRD patterns of pristine GF and GF@IZO showed no obvious diffraction peaks, confirming that both pristine GF and GF@IZO were amorphous. For GF@IZO after cycling in Zn–Zn symmetrical cell, the XRD pattern showed significant signals of indium metal (JCPDF no. 96‐901‐1600) and zinc metal (JCPDF no. 96‐153‐8015) (Figure 1e). This observation indicated that the black substances likely a In–Zn interface layer consists of metallic indium and metallic zinc. Scanning electron microscopy (SEM) of the cross‐section of the interface layer revealed the presence of a ∼12 µm dense region that distinguished the glass fibers and Zn anode. EDS mappings showed that this region was composed of indium and zinc, further confirming the composition of the interface layer (Figure 1f). Meanwhile, SEM of the interface clearly revealed that multiple layers encircled a glass fiber. Energy dispersive X‐ray spectrometer (EDS) mappings indicated that indium was coated on the glass fiber, and the surrounding layers consisted of substances where indium and zinc co‐existed, which corresponded to In–Zn interface layer (Figure 1g). According to the binary phase diagram of zinc with indium (Figure S6), indium may not form an alloy with zinc, implying that the black substances constitute an In–Zn hybrid interface composed of metallic indium and metallic zinc.

To characterize the valence state dynamics of In and Zn within the GF@IZO system, High‐resolution X‐ray photoelectron spectroscopy (XPS) was conducted at the conclusion of each charge/discharge cycle during the initial 2 cycles (Figure 1h). Characteristic signals corresponding to both In^0^ and Zn^0^ were successfully detected, and these findings align with the XRD and SEM results. Quantitative valence proportion analysis (bottom panels) further quantifies these trends (Figure 1i): the fractions of Zn^0^ (relative to total Zn) and In^0^ (relative to total In) both increase continuously with cycling time. These results confirm the synchronous metallic Zn deposition and In^3+^ reduction at the interface, which underpin the formation of the In–Zn hybrid interface and the dendrite‐suppression performance of GF@IZO. Meanwhile, the XRD patterns and the relative intensity ratio of In/Zn(101) are consistent with the above results (Figure S7).

The formation of a dense interface between the separator and anode was rarely observed. The significant morphology change in Zn deposit at the In–Zn hybrid interface might be attributed to the suppression of HER and the regulation of Zn^2+^ deposition, as shown in Figure 1j. For AZIBs with pristine GF, uneven electric field distribution causes two‐dimensional diffusion of Zn^2+^ on the Zn anode, leading to zinc dendrites formation [59]. Meanwhile, the coordination of Zn^2+^ with water triggers HER, and the generated OH^−^ reacts with electrolyte components to form byproducts like Zn_x_OTf(OH)_2x‐y_·H_2_O [60]. Additionally, the GF side is prone to chaotic ion migration and may cause performance degradation [44]. Conversely, after modifying the separator with IZO, an In–Zn interface layer forms in situ between the separator and Zn anode during cycling. The interface facilitates uniform Zn^2+^ migration and deposition through its uniform in‐plane electric field and high electrical conductivity. Furthermore, it suppresses HER and by‐product formation. In contrast to disordered zinc deposition and severe HER in pristine GF, the In–Zn hybrid interface provides more sufficient deposition sites for Zn^2+^, promotes uniform Zn^2+^ deposition within the interface layer, and effectively suppresses side reactions, thereby enhancing cycling performance.

### 2.2 Enhanced Stability With In–Zn Interface Layers

**FIGURE 2:**
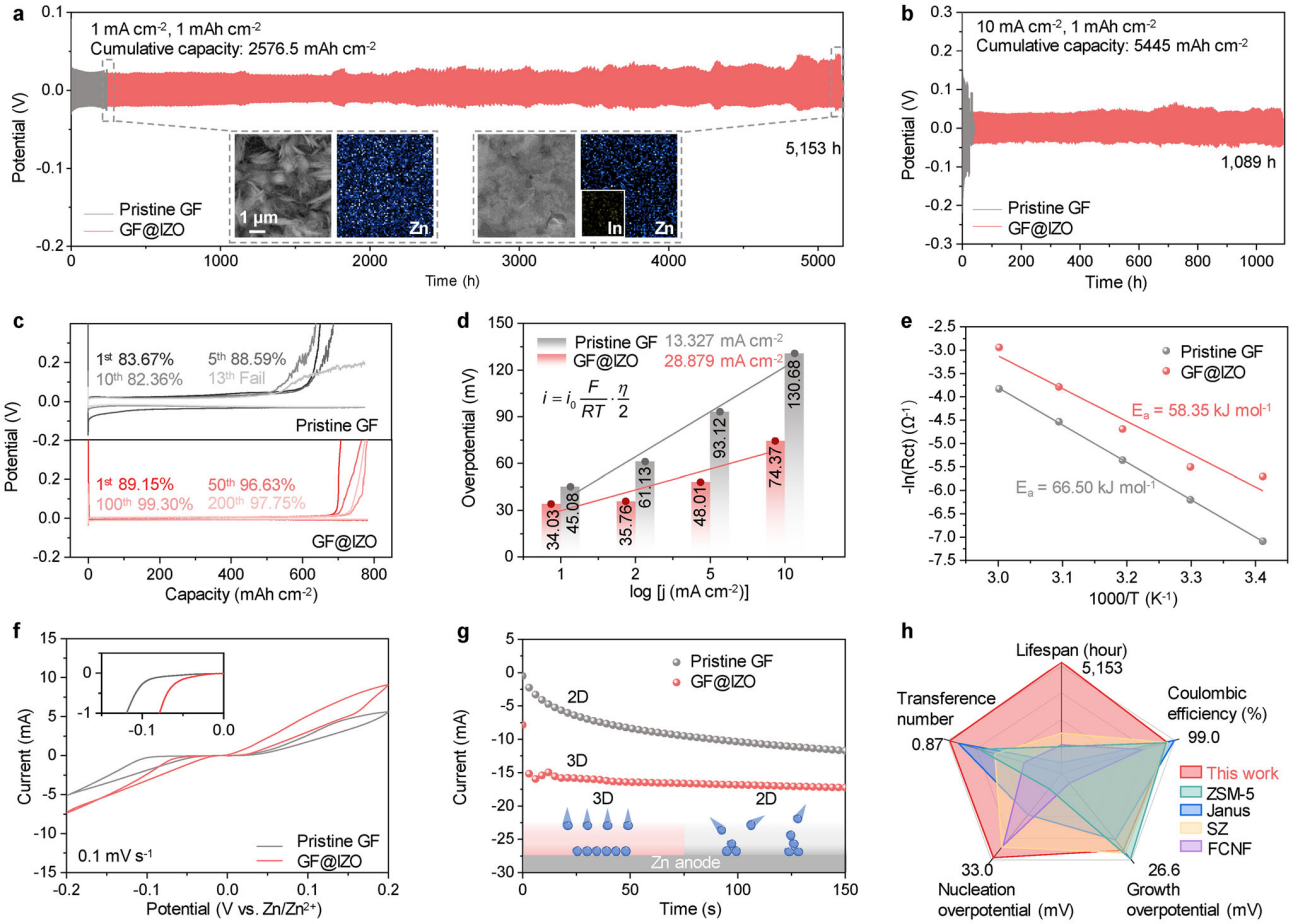
(a) Cycling performance of symmetrical cells at 1 mA cm^−2^@1 mAh cm^−2^. (b) Cycling performance of symmetrical cells at 10 mA cm^−2^@1 mAh cm^−2^. (c) The corresponding charge–discharge curves of Zn‐Ti half cells at 1 mA cm^−2^@1 mAh cm^−2^ with GF and GF@IZO. (d) Corresponding exchange current densities of GF and GF@IZO. (e) Arrhenius curves and the activation energies. (f) CV profiles of symmetric cells using GF and GF@IZO at 1 mV s^−1^. (g) CA profiles. (h) Comparison of lifespan, coulombic efficiency, growth overpotential, nucleation overpotential, and transference number of GF@IZO with representative peer Zn anodes.

Cycling stability of Zn–Zn symmetrical cells was assessed at 1 mA cm^−2^ and 1 mAh cm^−2^ (Figure 2a and Figure S8). The cell with pristine GF suffered a sudden voltage drop after 224 h, which was ascribed to a short circuit caused by the growth of Zn dendrites. Notably, the cell with GF@IZO sustained stable cycling for 5133 h, delivering a cumulative capacity of 2576.5 mAh cm^−2^. Postcycling SEM and EDS mapping of the cleaned Zn anode further validate this behavior, and the corresponding optical pictures in Figure S9: the pristine GF counterpart exhibits extensive dendritic Zn deposits, while the GF@IZO counterpart displays a smooth, uniform surface. EDS analysis confirms the distribution of In and Zn at the interface, implying that the in‐situ formed In–Zn hybrid interface acts as a protective interphase to suppress dendrites growth.

The more rapid battery failure is observed at higher current densities and cycling capacities (Figure 2b and Figure S10). Specifically, the cells with pristine GF experienced short circuits at ∼26 h (5 mA cm^−2^ and 5 mAh cm^−2^) and ∼40 h (10 mA cm^−2^ and 1 mAh cm^−2^), attributed to severe dendrite growth driven by nonuniform electric fields and depleted Zn^2+^ concentrations between separator and Zn anode. Remarkably, GF@IZO enables steady cycling at ∼400 h (5 mA cm^−2^ and 5 mAh cm^−2^) and ∼1100 h (10 mA cm^−2^ and 1 mAh cm^−2^), demonstrating robust tolerance to high current density cycling.

The Zn‐Ti half‐cells were assembled to investigate the reversibility of Zn deposition. At a current density of 1 mA cm^−2^, the Zn‐Ti half‐cells with GF@IZO operated stably for over 600 cycles with an average CE of 97.18%, exhibiting high reversibility and stable plating/stripping behaviors. By contrast, the CE of the Zn‐Ti half‐cells with GF exhibited fluctuating CE, dropping to only 82.36% after a mere 10 cycles (18 h), which was attributed to its poor reversibility (Figure 2c and Figure S11).

The cycling stability advantage of GF@IZO was further evaluated by galvanostatic tests on symmetrical cells (Figure S12). The rate capability of the symmetrical cells with GF and GF@IZO was tested at different current densities from 1 to 1000 mA cm^−2^ under a fixed areal capacity of 1 mAh cm^−2^. The results show that a battery with GF@IZO can achieve high stability at different current densities, exhibiting more excellent rate performance and lower voltage hysteresis. Further, we elected the symmetrical cells with GF@IZO had a higher value of exchange current density. The symmetrical cell with GF@IZO has a high *i*_0_ of 28.879 mA cm^−2^, nearly 2.2 times greater than that of GF (13.327 mA cm^−2^). The result demonstrates that the GF@IZO substantially enhances the kinetics of Zn^2+^ deposition [61]. Specifically, an elevated exchange current density corresponds to a larger critical nucleation radius during the initial nucleation stage, thereby facilitating the formation of a uniform and compact zinc deposition [62].

To analyze the effect of GF@IZO on the Zn plating and stripping reaction kinetics, we conducted EIS measurements on GF and GF@IZO symmetrical cells at different temperatures (Figure S13) and calculated the activation energy according to the Arrhenius equation (Figure 2e). It is obvious that the cells with the GF@IZO exhibit lower impedance under different temperatures, leading to depressed Arrhenius activation energy (E_a_ = 58.35 kJ mol^−1^) compared to that of GF (E_a_ = 66.50 kJ mol^−1^). These results demonstrate the critical role of In–Zn hybrid interface in Zn^2+^ desolvation and deposition processes.

To study the reversibility and electrochemical kinetics of a symmetrical cell with GF and GF@IZO during the plating/stripping process, CV tests are performed at a scan rate of 0.1 mV s^−1^. The cell with GF@IZO possesses a higher maximal current density and larger integrated peak area than GF, underscoring the improved kinetics and interfacial activity for zinc deposition endowed by In–Zn hybrid interface (Figure 2f).

Besides, the chronoamperometry curves (CA) (Figure 2g) show that, in sharp contrast to the continuous increase of the response current in the GF battery, the response current in the GF@IZO battery stabilizes quickly after applying an overpotential of −150 mV, testifying that the In–Zn interface layer can efficiently suppress the 2D Zn^2+^ diffusion within the surface between the separator and Zn anode. As a result, the GF@IZO is supposed to promote the multisite nucleation and uniform deposition of Zn^2+^. Ultimately, GF@IZO demonstrated comprehensive performance, surpassing relevant reported modification of separator in key metrics including lifespan, coulombic efficiency, deposition overpotential, nucleation overpotential, and Zn^2+^ transference number (Figure 2h and Table S1).

### 2.3 Stabilization Mechanism for In–Zn Interface Layers

**FIGURE 3:**
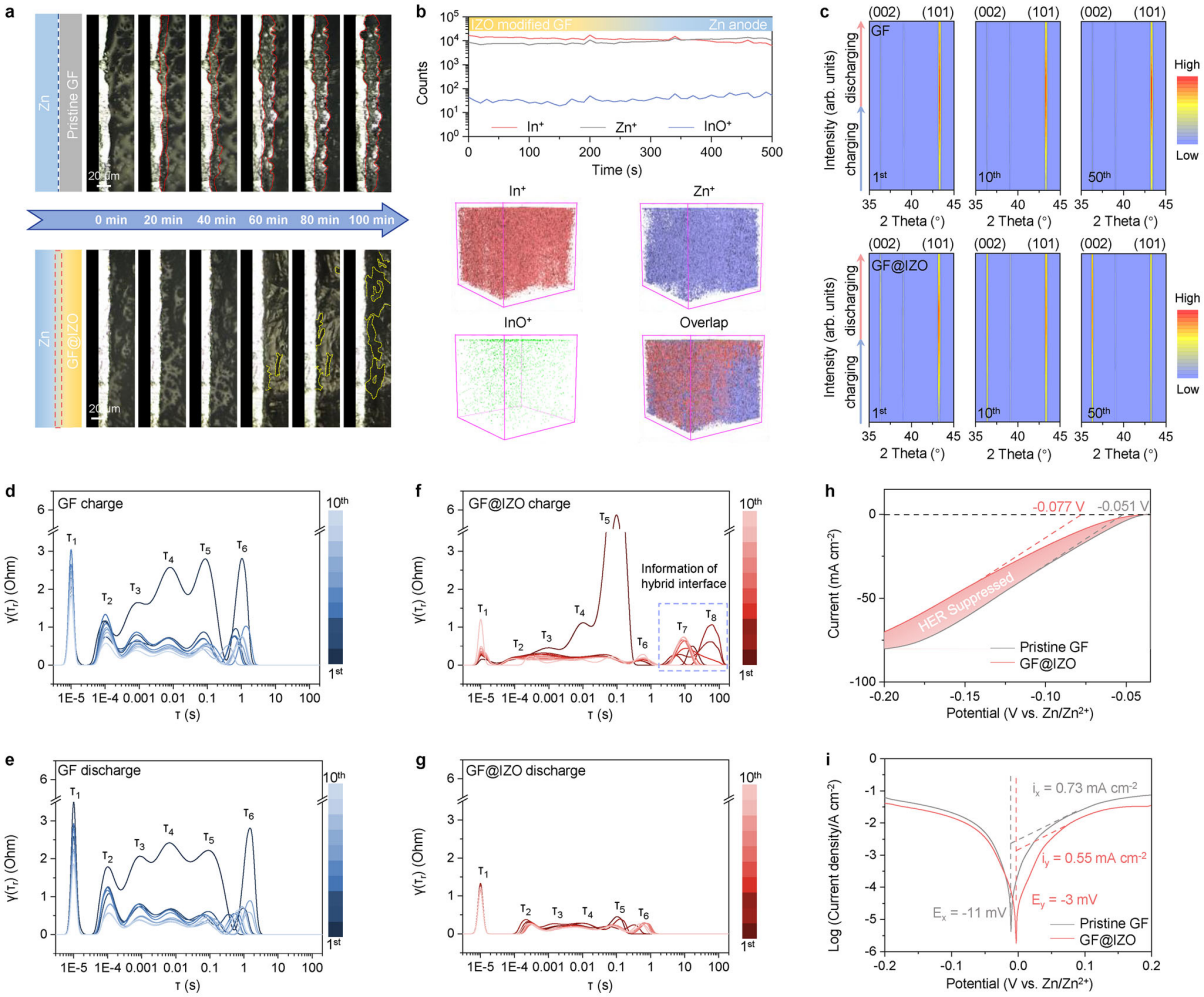
Relevant test of pristine GF and GF@IZO. (a) In situ observation of In–Zn interface layer between the Zn anode and pristine GF/GF@IZO at 1 mA cm^−2^ (scale bar: 20 µm). (b) Corresponding ToF‐SIMS 3D spectra and images with the In–Zn interface layer. (c) In situ XRD of Zn anode at first cycle, 10th cycle,50th cycle for GF and GF@IZO. DRT analysis of in situ EIS during Zn plating on (d,e) GF and (f,g) GF@IZO. (h) LSV for the corrosion. (i) Tafel plots for describing corrosion.

To elucidate the formation mechanism of the In–Zn interface layer, in situ optical microscopy was employed to monitor the morphological evolution during galvanostatic cycling at 1 mA cm^−2^ (Figure S14). For pristine GF, initially, the electrolyte infiltrates the separator. After 20 min, zinc metal deposition is observed on the surface of the Zn anode, and the deposited zinc becomes increasingly uneven over time. For GF@IZO, the separator also becomes fully infiltrated with electrolyte initially. Notably, after 20 min, no obvious zinc metal deposition is detected on the surface of the Zn anode. By 60 min, as the electrolyte spreads uniformly, the IZO‐coated glass fibers are exposed, exhibiting a bright appearance under microscopy, with distinct black areas forming on the anode surface. Also, some zinc metal begins to appear on the glass fibers. At 80 min, the black areas gradually expand. By 100 min, the black areas continue to expand and become closely adjacent to the zinc deposition areas. These observations clearly demonstrate that the IZO‐coated glass fibers provide abundant nucleation sites for zinc metal deposition. The black substance originates preferentially on the anode side, maintaining a distinct boundary with the zinc deposition region throughout the process (Figure 3a).

The Time‐of‐Flight Secondary Ion Mass Spectrometry (ToF‐SIMS) revealed the distribution of elements within the interface layer after many cycles (Figure 3b and Figure S15). Distinct concentration gradients demonstrated an asymmetric concentration gradient: In^+^ species exhibited preferential enrichment near the GF@IZO interface (∼70% intensity relative to bulk), while Zn^+^ species concentrated toward the Zn anode surface. This further indicates that the hybrid layer is composed of two elemental species: indium and zinc, which is consistent with previous reports [63, 64]. Moreover, to ensure an adequate indium source for the formation of the interface layer, the IZO coating was deliberately applied in excess, as confirmed by the InO^+^ signal intensity. 3D ion mapping demonstrated intimate intermixing of In^+^ and Zn^+^ species, with In^+^ penetrating progressively toward the Zn anode. This gradient‐confirmed interdiffusion confirmed the distribution of elements within the In–Zn interface layer.

The inherent pore structure of pristine GF typically induces localized electric field concentration, resulting in significant field inhomogeneity [65]. This inhomogeneity is mitigated by introducing a conductive layer onto the separator surface [66]. The conductive layer establishes an equipotential surface, thereby homogenizing the electric field distribution between the separator and the Zn anode. This uniform electric field, in conjunction with the zincophilic coating, promotes uniform deposition of Zn^2+^. COMSOL Multiphysics simulations elucidated the electric field distribution of pristine GF and the In–Zn interface layer (Figure S16). Compared with the surrounding framework, pristine GF exhibited pronounced electric field intensification at its pores. Such preferential Zn deposition evolves into Zn dendrites through a self‐amplification mechanism, eventually causing the internal short circuit of cells. In contrast, following the in situ formation of an In–Zn interface layer between GF and Zn anode, a homogeneous electric field emerges owing to the equipotential surface characteristics of the layer. Significantly, the field enhancement is confined within the In–Zn interface layer. The zincophilic property of the In–Zn interface layer facilitated the generation of a uniform Zn^2+^ flux under the homogeneous electric field, enabling smooth Zn^2+^ deposition on the Zn anode. Concurrently, a high zincophilic layer of the In–Zn interface layer induces synchronous zinc deposition on the Zn anode and separator [67, 68]. During cycling, zinc is deposited from the Zn anode and separator to form a dense morphological structure.

As visualized in Figure 3c, we systematically tracked the crystalline orientation evolution of the Zn anode via in situ XRD across cycles. The upper panel corresponds to cells employing GF separators, and the lower panel to those with GF@IZO separators. Diffraction peaks at 36.3° and 43.4° index to the (002) and (101) planes of Zn, respectively, and the color scale denotes diffraction peak intensity. For cells with GF, the diffraction peak intensity distribution grows increasingly heterogeneous with cycling. After 10 cycles, the intensity ratio of (002) to (101) deviates noticeably from its initial state. By 50 cycles, the peaks broaden slightly alongside uneven intensity fluctuations. This evolution signals progressive disorder in Zn crystalline orientation, consistent with nonuniform deposition induced by dendrite formation and subsequent structural deterioration of the anode surface. In contrast, cells with GF@IZO, corresponding to the lower panel, exhibit highly stable diffraction behavior over 50 cycles. Zn (002) becomes the dominant position at the ratio of (002) and (101). Such consistent crystalline orientation evolution directly corroborates the regulatory effect of GF@IZO. The tailored electric field distribution, simulated earlier, enhances Zn^2+^ deposition kinetics to facilitate homogeneous Zn nucleation and growth, while sustaining a well‐ordered crystalline arrangement of the Zn layer. This effect mitigates the structural disorder of the anode. This in situ XRD evidence provides direct crystallographic support for the long‐term stability of Zn deposition enabled by GF@IZO, aligning closely with the performance implications derived from our prior electric field simulations.

The critical moment when dendrites begin to form is termed Sand's time. It can be inferred that decreasing the transference number of anions (t_a_) would prolong Sand's time, thereby mitigating dendritic growth [69, 70]. Notably, unlike the formation of a dense solid‐electrolyte interphase on Li metal anodes in organic electrolytes, anions in commonly used Zn salts for aqueous electrolytes (e.g., SO_4_^2−^, OTf^−^) tend to generate loosely bound deprotonated hydroxyl species due to spontaneous water splitting, which exacerbates anode corrosion [71]. Therefore, reducing diffusible anions in aqueous electrolytes is essential not only to suppress dendrite formation but also to minimize the generation of undesirable byproducts. Significantly, the Sand's time of GF@IZO is substantially longer than that of GF.

It has been reported in the past that In_2_O_3_ is soluble in acidic solutions [72, 73] and the pH value of Zn(OTf)_2_ electrolyte is 3–4 (Figure S17). In order to confirm the solubility of In_2_O_3_ in Zn(OTf)_2_ electrolyte, we immersed the GF@IZO in the Zn(OTf)_2_ electrolyte for 48 h(Figure S18), which shows that In_2_O_3_ is slightly soluble in the electrolyte. In the Zn(OTf)_2_ electrolyte, the dissolved In^3+^ are extremely easy to form complexes with OTf^−^ (In^3+^ + 3OTf^−^ ⇌ In(OTf)_3_) [74]. Besides, it is well known that In^3+^ has a stronger oxidation than that of Zn^2+^, which implies the In^3+^ are preferentially reduced to indium metal [75]. Further, to confirm the specific effect of In_2_O_3_ in IZO, we analyzed the GF@In_2_O_3_ and the surface composition changes on the separator before and after cycling, which were compared using XPS (Figure S19). Significant signals corresponding to In and Zn were detected on the interface layer, consistent with the previous inferences. This confirms the generation of In–Zn interface layer during the cycling process.

To further resolve the dynamic evolution of interfacial processes during Zn plating/stripping, we perform in situ EIS measurement across the 10 cycles of symmetrical cells assembled with GF and GF@IZO (Figure S20). For the GF, the impedance is high due to the poor kinetics at the interface, and the impedance gradually decreases with the formation of the SEI at the interface, but it is still not very stable due to the complex physicochemical processes and side reactions at the interface. The initial impedance value of GF@IZO is lower than that of GF, while it only needs to be reduced to a stable impedance value after a short cycle, the kinetics are enhanced by the rapid formation of the interface layer, which is consistent with the results demonstrated by optical microscopy. At the same time, the Zn–Zn symmetrical cells also stably maintain a very low impedance, about half of that of GF, during subsequent cycles. The interfacial reduction demonstrated by the fitted circuits further corroborates the enhancement of interfacial kinetics, suggesting that the In–Zn interface layer can significantly improve interfacial stability.

Then, leverage distribution of relaxation times (DRT) to deconvolve the underlying interfacial processes. The results of DRT analysis are shown in Figure 3d–g, which can be mainly split in five peaks: Ohmic impedance (R_ohm_, τ_1_ ≈ 1 × 10^−5^ s), Zn^2+^ adsorption (R_ad_, τ_2_ ≈ 1 × 10^−4^ s, τ_3_ ≈ 2 × 10^−3^ s), Zn^2+^ migration at the electrode surface (R_mig_, τ_4_ ≈ 5 × 10^−2^ s), charge transfer impedance of Zn^2+^, (R_ct_, τ_5_ ≈ 0.4 s) and Zn^2+^ diffusion in the electrolyte (R_diffu_, τ_6_ ≈ 3.8 s) [76]. For the GF cell, charging profiles exhibit pronounced fluctuations across five resolved relaxation peaks over the 10 cycles. The R_ohm_ shows a modest rise from ∼0.5 to ∼0.8 Ω as charging proceeds, while the charge transfer peak (τ_5_ ≈ 0.4 s) undergoes a fold drop in impedance (from ∼2.0 Ω to ∼1.0 Ω) during plating, during discharging, diffusion associated peaks display irregular broadening, with varying between∼1.5 Ω and ∼2.5 Ω across cycles signaling heterogeneous Zn^2+^ transport dynamics at the unmodified GF interface. In contrast, the GF@lZO cell exhibits far more stable DRT behavior over the 2–9 cycles. The newly emerged peaks (τ_7_ and τ_8_) corresponding to the information of In–Zn hybrid interface. These quantitative DRT trends directly corroborate the regulatory role of the IZO modification layer. The information of the hybrid interface corresponds to controlled Zn^2+^ transport within the IZO layer, while suppressed impedance fluctuations for charge‐transfer and surface migration processes reflect homogenized Zn^2+^adsorption/migration kinetics across the first 10 cycles.

To verify the theoretical modification effect of GF@IZO, a series of electrochemical tests on GF@IZO and GF were carried out. The stabilized interface pH is thought to be related to the suppression of water decomposition as studied by the linear sweep voltammetry (LSV). Figure 3h displayed the LSV curves of the Zn–Zn symmetrical cells with GF@IZO. It was found that GF@IZO exhibited a lower current density for H2 evolution between −0.02 V and −0 V and an obvious negative shift of HER on‐set potential (−0.077 V vs. standard Zn electrode) than bare Zn (−0.051 V vs. standard Zn electrode), reflecting a delayed HER of GF@IZO. The onset potential of Zn deposition was reduced by 26 mV, which benefited from the suppressed deposition polarization by functional layer‐assisted desolvation [77]. In addition, the protective effect of the separators on the Zn anode was analyzed by linear polarization test (Figure 3i). The results show that Zn–Zn symmetrical cells using the GF@IZO exhibits a markedly smaller corrosion current density (0.55 mA cm^−2^) than that using the GF (0.73 mA cm^−2^), indicating that the IZO coating has a significant inhibition of the interfacial corrosion reactions. Collectively, these electrochemical results underscore that the In–Zn hybrid interface exerts a synergistic suppression effect on detrimental interfacial side reactions of the Zn anode, which hinders HER by inhibiting water splitting, alleviates deposition polarization via enhanced desolvation, and mitigates interfacial corrosion.

### 2.4 Electrochemical Performance of VO_x_‐Zn Cells

**FIGURE 4:**
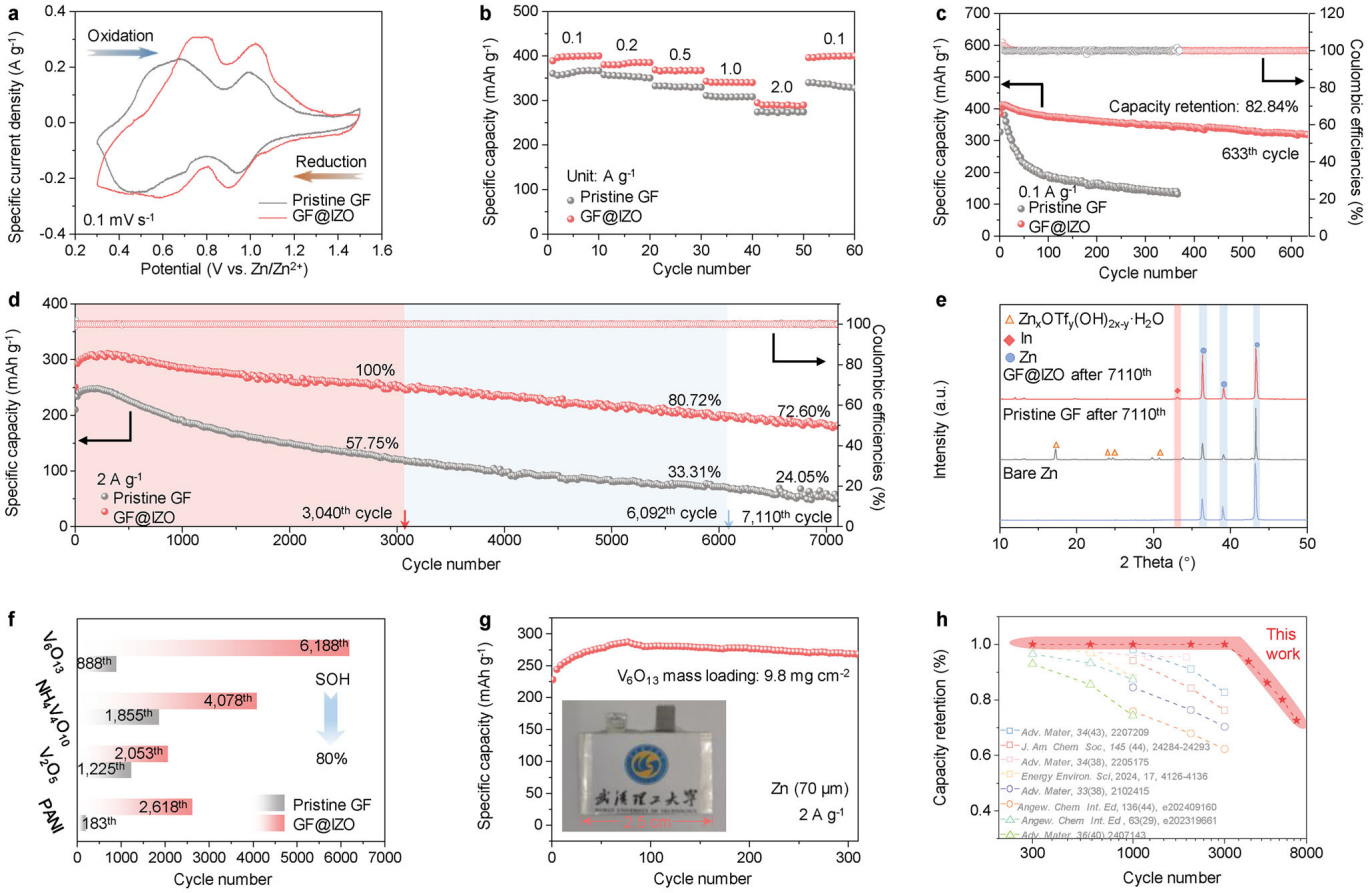
(a) CV curves of Zn||V_6_O_13_ full cells using different separators. (b) Rate performance for V_6_O_13_‐GF@IZO‐Zn and V_6_O_13_‐GF‐Zn. Cycling performance of the full cells at (c) 0.1 A g^−1^, (d) 2 A g^−1^. (e) XRD pattern of Zn anode surface after full cells cycling over 7110 cycles. (f) Number of cycles turns for different cathodes with GF@IZO and GF when the SOH deteriorated to 80%. (g) The long cycling performance of the pouch cell at 2 A g^−1^. (h) The comparison of cyclic performance with other reported Interface engineering.

Vanadium‐based materials have found extensive applications in AZIBs. Among them, V_6_O_13_ [78] was prioritized in research due to the inherent stability of V^4+^ and the high capacity contribution from V^5+^, prompting our selection of V_6_O_13_ as the cathode material. The as‐synthesized samples were characterized by SEM, XRD, and XPS, which confirmed the successful synthesis of V_6_O_13_ (Figure S21). The structure of full cells is shown in Figure S22. The cyclic voltammetry (CV) plots of the full cells display two reduction/oxidation peaks (Figure 4a) that are ascribed to the valence change of V^5+^ to V^4+^ and V^4+^ to V^3+^ associated with the Zn^2+^ insertion/extraction [79].

The discharge capacities of GF@IZO full cells with the V_6_O_13_ cathode were 388.83, 380.34, 368.61, 343.08, and 294.54 mAh g^−1^ at current densities of 0.1, 0.2, 0.5, 1, and 2 A g^−1^, respectively (Figure 4b). After cycling at varying current densities, the discharge capacity of the GF full cells decayed from 360.27 to 328.12 mAh g^−1^. In contrast, the discharge capacity of the GF@IZO full cells recovered to a high value of 396.1 mAh g^−1^, indicating that enhanced Zn^2+^ transport kinetics significantly improve the rate performance of the cells. The degradation of layered V‐based materials in AZIBs is usually amplified at a moderately low current density [80]. A current density of 0.1 A g^−1^ was chosen as the critical test condition. The GF full cells exhibited an initial discharge capacity of 327.61 mAh g^−1^ at 0.1 A g^−1^ (Initial absolute capacity: 0.39 mAh) (Figure 4c). But the capacity rapidly declined to 191.83 mAh g^−1^ after 100 cycles, retaining only 58.55% of the initial capacity. In contrast, GF@IZO full cells delivered an initial discharge capacity of 385.16 mAh g^−1^ and maintained stable cycling for 633 cycles with a CE of 99.99% and 82.84% capacity retention (Initial absolute capacity: 0.46 mAh). Furthermore, using an H‐type cell combined with ICP and XRD measurements, we find that the capacity of the pristine GF cell decays rapidly. The GF@IZO separator only provides a physical barrier against the vanadium ion shuttling effect, and it mainly stabilizes the battery system by suppressing side reactions (Figure S23). Moreover, GF@IZO full cells achieved over 7000 cycles at 2 A g^−1^ (Initial absolute capacity: 0.30 mAh) with a low‐capacity fading rate of 0.004% per cycle, significantly far outperforming reference batteries (Initial absolute capacity: 0.25 mAh) (Figure 4d). These results explicitly demonstrate that the GF@IZO substantially extends the cycle life of the Zn‐based cell.

To elucidate the structural origin of the enhanced cycling stability, XRD is conducted on the Zn anode harvested from the cells after 7110 cycles, with the patterns shown in Figure 4e. The XRD profile of the Zn anode paired with GF@IZO displays sharp, well‐defined peaks that are exclusively indexed to metallic Zn (consistent with standard Zn diffraction features), with no detectable impurity phases, indicating that the structure of the anode and compositional integrity are well preserved during cycling. Conversely, the XRD patterns of anodes from the pristine GF cells exhibit weak but distinct additional peaks, which can be attributed to anode corrosion byproducts. This structural contrast directly links the superior cycling performance of the GF@IZO cell to the effective suppression of interfacial parasitic reactions, which prevents the accumulation of detrimental byproducts and preserves the active Zn anode.

To evaluate the cycling stability of other cathodes, the GF@IZO was also matched with cathodes of NH_4_V_4_O_10_, V_2_O_5_, and PANI. The full cells were tested at a current density of 2 A g^−1^ until their state of health (SOH) decayed to 80% of the initial value [81]. For NH_4_V_4_O_10_, the lifespan increases from 1855th to 4078th. For commercial V_2_O_5_, the lifespan increases from 1225th to 2053th. For PANI, the lifespan increases from 183th to 2,618th. (Figure 4f and Figure S24). The results indicated that the mechanism of In–Zn interface layer is applicable across diverse cathode materials. Notably, a significant improvement in self‐discharge behavior was observed upon the in‐situ formation of In–Zn interface layer. The full cell using the GF@IZO could still hold 92.05% of its original capacity, surpassing that using GF (88.11%), which indicated that the In–Zn interface layer effectively improved the stability of various cathodes (Figure S25). To further validate the role of the in situ In–Zn interface layer in other electrolytes, we selected ZnSO_4_ with the same concentration as the 3 mol/L Zn(OTf)_2_ electrolyte for comparative analysis (Figure S26). For Zn‐Ti half cells, GF@IZO cells showed a higher average CE compared to GF. Also, the GF@IZO revealed better stability in Zn–Zn symmetrical cells and VO_x_ full cells. The comparison with different cathodes and electrolytes demonstrated that the modified GF@IZO separator could effectively enhance the performance of the battery.

To assess the viability of the GF@IZO in larger‐scale applications, a pouch‐type GF@IZO full cell (2 × 3 cm^2^) was assembled, as shown in Figure S27. The pouch cell exhibited an open circuit voltage of 1.31 V and maintained stable operation for 1000 cycles with over 60% capacity retention (Figure 4g). Figure 4h compared the capacity retention and cycle number of this work with recent interface engineering studied in AZIBs. This work retained 100% capacity at 3000 cycles, whereas other relevant works show degradation, highlighting its significantly longer cycle life (Table S2).

## 3 Conclusions

This work presented a separator engineering strategy with an in‐situ formation of a conductive and zincophilic In–Zn interface layer, offering a solution to suppress Zn dendrite growth and HER in AZIBs. Through systematic screening of In_2_O_3_ and its derivates, IZO was selected as the activator due to its intrinsic electrical conductivity and zincophilic characteristics, which enabled the formation of an In–Zn interface layer during cycling. Theoretical simulations revealed that the conductive In–Zn interface layer homogenizes the interfacial electric field, mitigating local field concentration around separator pores and guiding uniform Zn^2+^ deposition. This role of electrical conductivity and zincophilicity established a continuous ionic conductive pathway between the Zn anode and separator, effectively suppressing dendrite growth and HER by promoting ordered Zn plating/stripping kinetics. As a result, the Zn–Zn symmetrical cell showed a stable long lifespan over 5000 h at 1 mA cm^−2^ and 1 mAh cm^−2^. For V_6_O_13_‐Zn full cells, at 0.1 A g^−1^, a capacity of 413.17 mAh g^−1^ was obtained, and the capacity retention reacheed 82.24% after 633 cycles. Even at a high current density of 2 A g^−1^, the cells maintained 80.72% capacity retention over 6000 cycles, highlighting superior rate capability and structural durability. We note that this work focuses on understanding the fundamental properties of indium‐based oxide‐modified separators for AZIBs, rather than pursuing immediate practical applications. Commercializing a new separator technology remains a formidable challenge, as it requires the holistic evaluation of numerous interrelated parameters. Indium metal and its oxides indeed exhibit intriguing characteristics in terms of reaction kinetics and electrochemical reversibility; however, indium is a rare and costly element, which inevitably increases the overall battery cost. Fortunately, cost is not the sole determinant of a battery technology's commercial viability, and several expensive elements are already integrated into commercial battery systems. For instance, zinc–silver oxide batteries employ the costly silver element as a cathode component, while nickel–metal hydride batteries utilize expensive rare earth elements in their anode formulations. Following this line of reasoning, indium‐based oxide‐modified separators may not be suitable for large‐scale energy storage applications, but they could find niche utility in high‐end markets that demand exceptional performance and can accommodate relatively high costs, such as miniature devices, wearable technologies, and aerospace exploration systems.

## Conflicts of Interest

The authors declare no conflicts of interest.

## Supporting information




**Supporting File**: advs74972‐sup‐0001‐SuppMat.pdf.

## Data Availability

The data that support the findings of this study are available from the corresponding author upon reasonable request.

## References

[1] W. Yin , H. Xu , and J. Liu , “Advances in Aqueous Metal‐Sulfur‐Based Batteries with Conversion Mechanism,” Advanced Functional Materials 36 (2026): 12784, 10.1002/adfm.202512784.

[2] H. Xu , W. Yang , M. Li , et al., “Advances in Aqueous Zinc Ion Batteries based on Conversion Mechanism: Challenges, Strategies, and Prospects,” Small 20 (2024): 2310972, 10.1002/smll.202310972. 38282180

[3] H. Wang , L. Hu , H. Xu , and J. Liu , “Advances in Catalytic Host Cathodes for Aqueous Metal (Zn, Cu, Fe)‐Ion Batteries,” ACS Nano 19 (2025): 22645–22680, 10.1021/acsnano.5c05567. 40525942

[4] J. Liu , P. Xu , J. Liang , et al., “Boosting Aqueous Zinc‐ion Storage in MoS_2_ Via Controllable Phase” 389 (2020): 124405, 10.1016/j.cej.2020.124405.

[5] H. Xu , H. Liu , W. Yang , et al., “Enhanced Electrocatalytic Conversion of Tellurium with Mno Hollow Nanospheres Modified Hierarchical N‐doped Carbon Nanosheets in High‐performance Aqueous Zn‐te Battery,” Chemical Engineering Journal 485 (2024): 149825, 10.1016/j.cej.2024.149825.

[6] H. Zhai , H. Liu , Y. Zhang , et al., “Freestanding 1T MoS2@MXene Hybrid Film with Strong Interfacial Interaction for Highly Reversible Zinc Ions Storage,” Journal of Materials Science & Technology 188 (2024): 183–190, 10.1016/j.jmst.2023.12.015.

[7] H. Xu , P. Guo , C. Li , et al., “Heteroatoms Modulate the Copper Single Atom Catalytic Host Materials for Promoting the Redox Reaction in Aqueous Zinc‐Selenium Batteries,” Advanced Functional Materials 35 (2025): 2415016, 10.1002/adfm.202415016.

[8] H. Xu , S. Fan , H. Liu , et al., “The Modulation of Adsorption Balance Effect for Promoting Selenium Cathode Redox Reaction Kinetics in Aqueous Zinc‐Selenium Battery,” Energy Storage Materials 82 (2025): 104604, 10.1016/j.ensm.2025.104604.

[9] E. Molaei , M. M. Doroodmand , and R. Shaali , “Tartaric Acid as a Novel Additive for Approaching High‐Performance Capacity Retention in Zinc‐Ion Battery,” Scientific Reports 12 (2022): 13301, 10.1038/s41598-022-13897-5. 35922431 PMC9349178

[10] H. Ahn , D. Kim , M. Lee , and K. W. Nam , “Challenges and Possibilities for Aqueous Battery Systems,” Communications Materials 4 (2023): 37, 10.1038/s43246-023-00367-2.

[11] Y. Dai , R. Lu , C. Zhang , et al., “Zn^2+^‐Mediated Catalysis for Fast‐Charging Aqueous Zn‐Ion Batteries,” Nature Catalysis 7 (2024): 776–784, 10.1038/s41929-024-01169-6.

[12] A. Innocenti , D. Bresser , J. Garche , and S. Passerini , “A Critical Discussion of the Current Availability of Lithium and Zinc for Use in Batteries,” Nature Communications 15 (2024): 4068, 10.1038/s41467-024-48368-0. PMC11094038 38744859

[13] J. Luo , L. Xu , Y. Yang , et al., “Stable Zinc Anode Solid Electrolyte Interphase via Inner Helmholtz Plane Engineering,” Nature Communications 15 (2024): 6471, 10.1038/s41467-024-50890-0. PMC11291733 39085235

[14] Z. Yang , Y. Sun , S. Deng , et al., “Amphiphilic Electrolyte Additive as an Ion‐Flow Stabilizer Enables Superb Zinc Metal Batteries,” Energy & Environmental Science 17 (2024): 3443–3453, 10.1039/D4EE00318G.

[15] Z. Zheng , X. Zhong , Q. Zhang , et al., “An Extended Substrate Screening Strategy Enabling a Low Lattice Mismatch for Highly Reversible Zinc Anodes,” Nature Communications 15 (2024): 753, 10.1038/s41467-024-44893-0. PMC10810881 38272872

[16] C. Yang , P. Woottapanit , S. Geng , et al., “A Multifunctional Quasi‐Solid‐State Polymer Electrolyte with Highly Selective Ion Highways for Practical Zinc Ion Batteries,” Nature Communications 16 (2025): 183, 10.1038/s41467-024-55656-2. PMC11697030 39747185

[17] Y. Meng , M. Wang , J. Wang , et al., “Robust Bilayer Solid Electrolyte Interphase for Zn Electrode with High Utilization and Efficiency,” Nature Communications 15 (2024): 8431, 10.1038/s41467-024-52611-z. PMC11439932 39343779

[18] D. Kundu , B. D. Adams , V. Duffort , S. H. Vajargah , and L. F. Nazar , “A High‐Capacity and Long‐Life Aqueous Rechargeable Zinc Battery Using a Metal Oxide Intercalation Cathode,” Nature Energy 1 (2016): 16119, 10.1038/nenergy.2016.119.

[19] S. Wang , X. Guo , K. Huang , et al., “Cooperative Jahn‐Teller Effect and Engineered Long‐Range Strain in Manganese Oxide/Graphene Superlattice for Aqueous Zinc‐Ion Batteries,” Nature Communications 16 (2025): 5191, 10.1038/s41467-025-60558-y. PMC12137938 40467665

[20] Y. Mu , Z. Li , B. Wu , et al., “3D Hierarchical Graphene Matrices Enable Stable Zn Anodes for Aqueous Zn Batteries,” Nature Communications 14 (2023): 4205, 10.1038/s41467-023-39947-8. PMC10349079 37452017

[21] D. Ma , F. Li , K. Ouyang , et al., “An Electrochemically Driven Hybrid Interphase Enabling Stable Versatile Zinc Metal Electrodes for Aqueous Zinc Batteries,” Nature Communications 16 (2025): 4817, 10.1038/s41467-025-60190-w. PMC12102169 40410170

[22] Z. Wang , J. Huang , Z. Guo , et al., “A Metal‐Organic Framework Host for Highly Reversible Dendrite‐Free Zinc Metal Anodes,” Joule 3 (2019): 1289–1300, 10.1016/j.joule.2019.02.012.

[23] W. Zhang , S. Zhu , T. Yang , et al., “Hydrogen/Electron Amphiphilic Bi‐Functional Water Molecular Inactivator‐Assisted Interface Stabilization in Highly Reversible Zn Metal Batteries,” Angewandte Chemie International Edition 64 (2025): 202419732, 10.1002/anie.202419732. 39655630

[24] Y. Li , X. Zheng , E. Z. Carlson , et al., “In Situ Formation of Liquid Crystal Interphase in Electrolytes with Soft Templating Effects for Aqueous Dual‐Electrode‐Free Batteries,” Nature Energy 9 (2024): 1350–1359, 10.1038/s41560-024-01638-z.

[25] L. Liu , X. Wang , Z. Hu , et al., “Electric Double Layer Regulator Design through a Functional Group Assembly Strategy towards Long‐Lasting Zinc Metal Batteries,” Angewandte Chemie 63 (2024): 202405209, 10.1002/anie.202405209. 38712643

[26] J. Heo , D. Dong , Z. Wang , F. Chen , and C. Wang , “Electrolyte Design for Aqueous Zn Batteries,” Joule 9 (2025): 101844, 10.1016/j.joule.2025.101844.

[27] M. Liu , X. Li , M. Cui , et al., “Amorphous Organic‐Hybrid Vanadium Oxide for Near‐Barrier‐Free Ultrafast‐Charging Aqueous Zinc‐Ion Battery,” Nature Communications 15 (2024): 10769, 10.1038/s41467-024-55000-8. PMC11686002 39737938

[28] Y. Zhao , S. Guo , M. Chen , et al., “Tailoring Grain Boundary Stability of Zinc‐Titanium Alloy for Long‐Lasting Aqueous Zinc Batteries,” Nature Communications 14 (2023): 7080, 10.1038/s41467-023-42919-7. PMC10625522 37925505

[29] J. Cao , H. Wu , D. Zhang , et al., “In‐Situ Ultrafast Construction of Zinc Tungstate Interface Layer for Highly Reversible Zinc Anodes,” Angewandte Chemie International Edition 63 (2024): 202319661, 10.1002/anie.202319661. 38703353

[30] Z. Jiang , Y. Zhang , D. B. Ravnsbæk , et al., “An Adaptable Structure of Metal‐Organic Framework Glass Interlayer Enables Superior Performance in Aqueous Zinc‐Ion Batteries,” Advanced Materials 37 (2025): 2413167, 10.1002/adma.202413167. 39969417

[31] P. Xiao , H. Li , J. Fu , et al., “An Anticorrosive Zinc Metal Anode with Ultra‐Long Cycle Life over One Year,” Energy & Environmental Science 15 (2022): 1638–1646, 10.1039/D1EE03882F.

[32] Y. Zhao , S. Guo , M. Chen , et al., “Tailoring Grain Boundary Stability of Zinc‐Titanium Alloy for Long‐Lasting Aqueous Zinc Batteries,” Nature Communications 14 (2023): 7080, 10.1038/s41467-023-42919-7. PMC10625522 37925505

[33] Z. Wei , G. Qu , Z. Huang , et al., “Gradient Distribution of Zincophilic Sites for Stable Aqueous Zinc‐Based Flow Batteries with High Capacity,” Advanced Materials 36 (2024): 2414388, 10.1002/adma.202414388. 39543439

[34] G. R. Pastel , T. P. Pollard , Q. Liu , et al., “Designing Interphases for Highly Reversible Aqueous Zinc Batteries,” Joule 8 (2024): 1050–1062, 10.1016/j.joule.2024.02.002.

[35] J. Cao , D. Zhang , C. Gu , et al., “Modulating Zn Deposition via Ceramic‐Cellulose Separator with Interfacial Polarization Effect for Durable Zinc Anode,” Nano Energy 89 (2021): 106322, 10.1016/j.nanoen.2021.106322.

[36] Y. Dai , C. Zhang , J. Li , et al., “Inhibition of Vanadium Cathodes Dissolution in Aqueous Zn‐Ion Batteries,” Advanced Materials 36 (2024): 2310645, 10.1002/adma.202310645. 38226766 PMC11475447

[37] M. Zhang , C. Sun , G. Chen , et al., “Synergetic Bifunctional Cu‐In Alloy Interface Enables Ah‐Level Zn Metal Pouch Cells,” Nature Communications 15 (2024): 9455, 10.1038/s41467-024-53831-z. PMC11530701 39487128

[38] Y. Kim , Y. Park , M. Kim , J. Lee , K. J. Kim , and J. W. Choi , “Corrosion as the Origin of Limited Lifetime of Vanadium Oxide‐Based Aqueous Zinc Ion Batteries,” Nature Communications 13 (2022): 2371, 10.1038/s41467-022-29987-x. PMC9061739 35501314

[39] M. Kwon , J. Lee , S. Ko , et al., “Stimulating Cu–Zn Alloying for Compact Zn Metal Growth towards High Energy Aqueous Batteries and Hybrid Supercapacitors,” Energy & Environmental Science 15 (2022): 2889–2899, 10.1039/D2EE00617K.

[40] J. Zheng , Y. Deng , W. Li , et al., “Design Principles for Heterointerfacial Alloying Kinetics at Metallic Anodes in Rechargeable Batteries,” Science Advances 8 (2022): abq6321, 10.1126/sciadv.abq6321. PMC9635833 36332032

[41] Z. Cai , Y. Ou , B. Zhang , et al., “A Replacement Reaction Enabled Interdigitated Metal/Solid Electrolyte Architecture for Battery Cycling at 20 mA cm^−2^ and 20 mAh cm^−2^ ,” Journal of the American Chemical Society 143 (2021): 3143–3152, 10.1021/jacs.0c11753. 33595314

[42] K. Hu , X. Guan , R. Lv , et al., “Stabilizing Zinc Metal Anodes by Artificial Solid Electrolyte Interphase through a Surface Ion‐Exchanging Strategy,” Chemical Engineering Journal 396 (2020): 125363, 10.1016/j.cej.2020.125363.

[43] Y. Chai , X. Xie , Z. He , et al., “A Smelting–Rolling Strategy for Znin Bulk Phase Alloy Anodes,” Chemical Science 13 (2022): 11656–11665, 10.1039/D2SC04385H. 36320391 PMC9555725

[44] X. Lv , X. Gu , R. Tian , et al., “Artificial Solid Electrolyte Interphases Stabilized Zn Metal Anodes for High‐Rate and Long‐Lifespan Aqueous Batteries,” Electrochimica Acta 524 (2025): 146053, 10.1016/j.electacta.2025.146053.

[45] M. Zhang , S. Li , R. Tang , et al., “Stabilizing Zn/Electrolyte Interphasial Chemistry by a Sustained‐Release Drug Inspired Indium‐Chelated Resin Protective Layer for High‐Areal‐Capacity Zn//V_2_O_5_ Batteries,” Angewandte Chemie International Edition 63 (2024): 202405593, 10.1002/anie.202405593. 38716660

[46] J. Wu , L. Yang , S. Wang , et al., “Triple‐Functional Amorphous in_2_O_3_ Anode Protection Layer Design for High‐Performance Aqueous Zinc Ion Batteries,” Advanced Functional Materials 35 (2025): 2419492, 10.1002/adfm.202419492.

[47] Y. Meng , M. Wang , J. Xu , et al., “Balancing Interfacial Reactions through Regulating p‐Band Centers by an Indium Tin Oxide Protective Layer for Stable Zn Metal Anodes,” Angewandte Chemie International Edition 62 (2023): 202308454, 10.1002/anie.202308454. 37563746

[48] Y. Lv , M. Zhao , Y. Du , Y. Kang , Y. Xiao , and S. Chen , “Engineering a Self‐Adaptive Electric Double Layer on both Electrodes for High‐Performance Zinc Metal Batteries,” Energy & Environmental Science 15 (2022): 4748–4760, 10.1039/D2EE02687B.

[49] Z. Luo , Y. Xia , S. Chen , et al., “A Homogeneous Plating/Stripping Mode with Fine Grains for Highly Reversible Zn Anodes,” Energy & Environmental Science 17 (2024): 6787–6798, 10.1039/D4EE02264E.

[50] Y. Liang , D. Ma , N. Zhao , et al., “Novel Concept of Separator Design: Efficient Ions Transport Modulator Enabled by Dual‐Interface Engineering toward Ultra‐Stable Zn Metal Anodes,” Advanced Functional Materials 32 (2022): 2112936, 10.1002/adfm.202112936.

[51] H. Tang , H. Luo , G. Yu , et al., “Modulating Diffusion Kinetics and Interfacial Stability via in‐Situ Constructed Self‐Healing Interfaces for Highly Reversible Zinc Metal Anodes,” Angewandte Chemie International Edition 137 (2025): 202509622, 10.1002/anie.202509622. 40474477

[52] W. Du , E. H. Ang , Y. Yang , Y. Zhang , M. Ye , and C. C. Li , “Challenges in the Material and Structural Design of Zinc Anode towards High‐performance Aqueous Zinc‐Ion Batteries,” Energy & Environmental Science 13 (2020): 3330–3360, 10.1039/D0EE02079F.

[53] H. Yao , K. Yan , W. Li , et al., “Improved Lithium–Sulfur Batteries with a Conductive Coating on the Separator to Prevent the Accumulation of Inactive S‐Related Species at the Cathode–Separator Interface,” Energy & Environmental Science 7 (2014): 3381–3390, 10.1039/C4EE01377H.

[54] X. Xie , S. Liang , J. Gao , et al., “Manipulating the Ion‐Transfer Kinetics and Interface Stability for High‐Performance Zinc Metal Anodes,” Energy & Environmental Science 13 (2020): 503–510, 10.1039/C9EE03545A.

[55] M. Sun , G. Ji , and J. Zheng , “A Hydrogel Electrolyte with Ultrahigh Ionic Conductivity and Transference Number Benefit from Zn^2+^ “Highways” for Dendrite‐Free Zn‐MnO_2_ Battery,” Chemical Engineering Journal 463 (2023): 142535, 10.1016/j.cej.2023.142535.

[56] H. Peng , C. Wang , D. Wang , X. Song , C. Zhang , and J. Yang , “Dynamic Zn/Electrolyte Interphase and Enhanced Cation Transfer of Sol Electrolyte for all‐Climate Aqueous Zinc Metal Batteries,” Angewandte Chemie International Edition 62 (2023): 202308068, 10.1002/anie.202308068. 37400421

[57] J. Zhou , Y. Mei , F. Wu , et al., “Regulated Ion/Electron‐Conducting Interphase Enables Stable Zinc‐Metal Anodes for Aqueous Zinc‐Ions Batteries,” Angewandte Chemie International Edition 62 (2023): 202304454, 10.1002/anie.202304454. 37218359

[58] M. Yan , C. Xu , Y. Sun , H. Pan , and H. Li , “Manipulating Zn Anode Reactions through Salt Anion Involving Hydrogen Bonding Network in Aqueous Electrolytes with PEO Additive,” Nano Energy 82 (2021): 105739, 10.1016/j.nanoen.2020.105739.

[59] M. Zhang , W. Xu , X. Han , et al., “Unveiling the Mechanism of the Dendrite Nucleation and Growth in Aqueous Zinc Ion Batteries,” Advanced Energy Materials 14 (2024): 2303737, 10.1002/aenm.202303737.

[60] X. Yu , M. Chen , Z. Li , et al., “Unlocking Dynamic Solvation Chemistry and Hydrogen Evolution Mechanism in Aqueous Zinc Batteries,” Journal of the American Chemical Society 146 (2024): 17103–17113, 10.1021/jacs.4c02558. 38869216

[61] X. Yang , Y. Lu , Z. Liu , et al., “Facet‐Governed Zn Homoepitaxy via Lattice Potential Regulation,” Energy & Environmental Science 17 (2024): 5563–5575, 10.1039/D4EE00881B.

[62] H. Lu , D. Zhang , Q. Jin , et al., “Gradient Electrolyte Strategy Achieving Long‐Life Zinc Anodes,” Advanced Materials 35 (2023): 2300620, 10.1002/adma.202300620. 36946149

[63] S. Li , C. Sun , M. Zhang , et al., “An Integrated Tandem‐Structured Separator Enables Dual‐Enhanced Stable Interfaces for Long‐Cycle‐Life and High‐Areal‐Capacity Aqueous Zinc–Iodine Batteries,” Angewandte Chemie International Edition 64 (2025): 202506849, 10.1002/anie.202506849. 40525316

[64] Z. Lv , R. Tang , C. Sun , et al., “Inhibiting Cathode Dissolution and Shuttling of V–O Species Using a Polybenzimidazole Hydrogel Electrolyte for Durable High‐Areal‐Capacity Zn–V_2_O_5_ Batteries,” Energy & Environmental Science 18 (2025): 762–773, 10.1039/D4EE03857F.

[65] P. Tian , Y. Gao , S. Huang , et al., “Constructing Gradient Separator to Stabilize Bi‐Electrodes toward High‐Performance Zn Metal Batteries,” Advanced Energy Materials 14 (2024): 2401830, 10.1002/aenm.202401830.

[66] S. Liu , Q. Han , C. He , et al., “Ion‐Sieving Separator Functionalized by Natural Mineral Coating toward Ultrastable Zn Metal Anodes,” ACS Nano 18 (2024): 25880–25892, 10.1021/acsnano.4c09678. 39236748

[67] X. Chen , R. Zhang , R. Zhao , et al., “A “Dendrite‐Eating” Separator for High‐Areal‐Capacity Lithium‐Metal Batteries,” Energy Storage Materials 31 (2020): 181–186, 10.1016/j.ensm.2020.06.037.

[68] T. Foroozan , V. Yurkiv , S. Sharifi‐Asl , R. Rojaee , F. Mashayek , and R. Shahbazian‐Yassar , “Non‐Dendritic Zn Electrodeposition Enabled by Zincophilic Graphene Substrates,” ACS Applied Materials & Interfaces 11 (2019): 44077–44089, 10.1021/acsami.9b13174. 31674758

[69] P. Zou , Y. Sui , H. Zhan , et al., “Polymorph Evolution Mechanisms and Regulation Strategies of Lithium Metal Anode under Multiphysical Fields,” Chemical Reviews 121 (2021): 5986–6056, 10.1021/acs.chemrev.0c01100. 33861070

[70] Y. He , C. Wang , P. Zou , R. Lin , E. Hu , and H. L. Xin , “Anion‐Tethered Single Lithium‐Ion Conducting Polyelectrolytes through UV‐Induced Free Radical Polymerization for Improved Morphological Stability of Lithium Metal Anodes,” Angewandte Chemie International Edition 62 (2023): 202308309, 10.1002/ange.202308309. 37548104

[71] J.‐L. Yang , L. Liu , Z. Yu , et al., “Dielectric–Metallic Double‐Gradient Composition Design for Stable Zn Metal Anodes,” ACS Energy Letters 8 (2023): 2042–2050, 10.1021/acsenergylett.3c00367.

[72] S.‐Y. Han , G. S. Herman , and C. Chang , “Low‐Temperature, High‐Performance, Solution‐Processed Indium Oxide Thin‐Film Transistors,” Journal of the American Chemical Society 133 (2011): 5166–5169, 10.1021/ja104864j. 21417268

[73] W. Chou , Y. Shen , S. Yang , T. Hsiao , and L. Huang , “Recovery of Indium from the Etching Solution of Indium Tin Oxide by Solvent Extraction,” Environmental Progress & Sustainable Energy 35 (2016): 758–763, 10.1002/ep.12304.

[74] T. O. Ajiboye , O. J. Ajala , J. O. Adeyemi , and S. Dhibar , “Indium(III) Complexes: Application as Organic Catalyst, Precursor for Chalcogenides Nanoparticles and Starting Materials in the Industry,” Chemical Papers 78 (2024): 4605–4622, 10.1007/s11696-024-03411-8.

[75] D. Han , S. Wu , S. Zhang , et al., “A Corrosion‐Resistant and Dendrite‐Free Zinc Metal Anode in Aqueous Systems,” Small 16 (2020): 2001736, 10.1002/smll.202001736. 32567230

[76] M. Yan , C. Xu , Y. Sun , H. Pan , and H. Li , “Manipulating Zn Anode Reactions through Salt Anion Involving Hydrogen Bonding Network in Aqueous Electrolytes with PEO Additive,” Nano Energy 82 (2021): 105739, 10.1016/j.nanoen.2020.105739.

[77] P. Liang , J. Yi , X. Liu , et al., “Highly Reversible Zn Anode Enabled by Controllable Formation of Nucleation Sites for Zn‐Based Batteries,” Advanced Functional Materials 30 (2020): 1908528, 10.1002/adfm.201908528.

[78] Y. Dai , C. Zhang , J. Li , et al., “Inhibition of Vanadium Cathodes Dissolution in Aqueous Zn‐Ion Batteries,” Advanced Materials 36 (2024): 2310645, 10.1002/adma.202310645. 38226766 PMC11475447

[79] X. Wang and Y. Qin , Securing Vanadium by Ion‐Anchoring Separators for Long‐Lasting Aqueous Zinc‐Vanadium Batteries (2023), 10.21203/rs.3.rs-3062213/v1.

[80] C. Li , S. Jin , L. A. Archer , and L. F. Nazar , “Toward Practical Aqueous Zinc‐Ion Batteries for Electrochemical Energy Storage,” Joule 6 (2022): 1733–1738, 10.1016/j.joule.2022.06.002.

[81] J. Shi , T. Koketsu , Z. Zhu , et al., “In Situ p‐Block Protective Layer Plating in Carbonate‐Based Electrolytes Enables Stable Cell Cycling in Anode‐Free Lithium Batteries,” Nature Materials 23 (2024): 1686–1694, 10.1038/s41563-024-01997-8. 39223271

