# Supplementary material for: In Situ Formation of an In–Zn Interface Layer Enables Aqueous Zinc‐Ions Batteries with High Capacity Retention
Source: Adv Sci (Weinh). 2026 Apr 14;13(32):e74972. doi: 10.1002/advs.74972 (PMC13252613; doi:10.1002/advs.74972)
Supplement: Supplementary file 1 — Supporting File: advs74972‐sup‐0001‐SuppMat.pdf. [file ADVS-13-e74972-s001.pdf]

Supporting Information

In situ formation of an In-Zn hybrid layer between the separator and anode enables aqueous Zinc-ions batteries with high capacity retention

*Youwei Jiang, Jinghao Li, Jie Huang, Tan Qiu, Xiuwen Song, Fujun Deng, Yongxue Wang, YueMing, Ziping Wu, Qinyou An and Yaoguang Rong**

corresponding author: ygrong2022@outlook.com

Methods

Preparation of GF@IZO, GF@ITO and GF@In_2O_3 separator.

The IZO (In_2O_3: ZnO = 9:1) layer was coated onto a commercially available glass fiber (GF) separator using direct current magnetron sputtering system. The distance between the IZO target and GF as 10 cm. The sputtering time was set as 5, 10, 15, 20 and 25 min at the power of 150 W. The average loading mass of IZO layer was ~0.028 mg/cm^2 for sputtering time of 1 min. The GF@ITO and GF@In_2O_3 used the same approach.

Preparation of V_6O_{13} Cathode[1]: In the typical synthesis process, 1.2 g of V_2O_5 and 1.8 g of $H_2C_2O_4 \cdot 2H_2O$ were added to 40 mL of deionized (DI) water. The resulting mixture was then magnetically stirred at 75 °C for 1 h, yielding a dark blue solution. Subsequently, this solution was transferred into a 50 mL Teflon-lined autoclave and kept at 180 °C for 3 h. After the completion of the reaction, the autoclave was allowed to cool down to room temperature naturally. The resulting products were washed with ethanol and DI water, then dried at 60 °C overnight before being collected.

Preparation of NH_4VO_{10} Cathode[2]: NH_4VO_{10} was synthesized through a one-step hydrothermal approach. Specifically, 0.64 g NH_4VO_3 was mixed into deionized (DI) water (80 mL), heated to 60℃, and stirred. Then 1.16 g $H_2C_2O_4 \cdot 2H_2O$ was stirred into the solution until it finally turned to transparent dark green. Finally, the solution was placed in a 100 mL Teflon-lined autoclave and kept at 180℃ for 3h. The dark green NH_4VO_{10} powder was obtained by repeatedly rinsing with distilled water/alcohol and drying under a vacuum at 60 °C for 24 h. The material was marked as NVO.

Preparation of PANI Cathode[3]: 0.4 mL aniline monomer was dissolved in dichloromethane (40 mL) with stirring for 30 min to form A solution. 0.495 g manganese chloride tetrahydrate and 0.5 g ammonium persulfate (APS) were added into 40 mL of 0.5 M HCl and stirred for 30 min to form solution B. After that, the A and B solutions were mixed to obtain an aqueous/organic

stratification system with a clear interface. The reaction system was kept at 5 °C for 24 h. Afterward, the product was filtered, washed with deionized water and ethanol, and finally dried at 60°C for 12 h under vacuum.

Simulation[4]: With reference to previous research, the COMSOL electrical conduction models were adopted to conduct the simulation of the influence of the hybrid layer modified separator on the electric field distribution. The simulation was carried out in a rectangle area, the size of which is 120 μm by 100 μm. The pristine separator was modeled as a sieve plate with a thickness of 30 μm. The sieve plate is composed of rectangular channels with an aperture of 2.7 μm and a pore spacing of 5.0 μm. The conductor coating is modeled as a thin film with a thickness of 5.0 μm. The current between cathode and anode was set as $1.44e^{-7}$ A. The electrical conductivity of anode/cathode and separator was 5.0e7 and $1.0e^{-7}$ S m^{-1}, respectively. The electrical conductivity of hybrid layer and electrolyte was $1.0e^{5}$ and 5.00 S m^{-1}, respectively. In both the pristine separator and the modified separator, we assumed an electrolyte layer between the separator and the anode, rather than allowing them to be in direct contact. This is because the surfaces of the anode and the separator are not absolutely smooth, which would result in them being in partial rather than full contact, even under external pressure.

Electrochemical measurements. The working cathode of V_6O_{13} comprised active materials (70 wt%), acetylene black conductive additive (20 wt%), and polyvinylidene fluoride (PVDF) (10 wt%). The mixtures were dispersed in N-methylpyrrolidone (NMP), and then coated onto the Ti mesh (Φ = 12 mm) to make the cathodes. A piece of Zn foil with a thickness of 70 μm was punched into disks (Φ = 10 mm) as the anodes. The GF, GF@In_2O_3, GF@ITO and GF@IZO separator were punched into disks (Φ = 17 mm) to separate the cathode and anode, and 3 M $Zn(CF_3SO_3)_2$ aqueous solution was used as the electrolyte. All symmetrical and half-battery was assembled with CR-2032 type coin-cell. All batteries were assembled in the air atmosphere. Galvanostatic charge-discharge (GCD) cycling tests were performed on the NEWARE multichannel battery test system. The electrochemical impedance spectroscopy (EIS) of electrolyte, cyclic voltammetry (CV) profiles of Zn||Zn battery, linear sweep voltammetry (LSV), Tafel curves, chronoamperometry (CA) and the in-situ EIS of the batteries were recorded on an electrochemical workstation (CHI760E, China).

Ionic conductivities of the separators were measured using stainless steel as the electrodes and calculated through the following equation:

$$\sigma = \frac{d}{RS}$$

Where d represents the thickness of the separator, R represents the resistance according to EIS measurement, and S is the contact area

between the separator and electrodes.

The Zn^{2+} transference number ($t_{Zn^{2+}}$) was calculated by the following equation:

$$t_{Zn^{2+}} = \frac{I_s(\Delta V - I_0 R_0)}{I_0(\Delta V - I_s R_s)}$$

Where ΔV is the applied voltage (10 mV); I_0 and R_0 are the initial current and resistance, respectively. I_s and R_s are the steady-state current and resistance, respectively. I_s is the average value of the steady-state current.

The activation energy (E_a) of the zinc deposition was obtained by EIS measurements of Zn||Zn symmetric batteries at different temperatures and calculated by the following equation:

$$\frac{1}{R_{ct}} = A\exp\frac{E_a}{RT}$$

where R_{ct} is the charge-transfer resistance obtained from the EIS spectra, R is the gas constant, and T is the thermodynamic temperature.

The current ratio between the oxidation and reduction peaks is related to the reversibility of the conversion reaction according to the Randles–Sevcik equation:

$$I_p = 0.446nFAC\left(\frac{nFvD}{RT}\right)^{1/2}$$

where I_p is the peak current, n is the number of transferred electrons, A is the surface area of the working electrode, C is the concentration of the redox species, v is the scan rate, F is the Faraday constant, R is the universal gas constant, T is the temperature and D is the ion diffusion coefficient.

Theoretical interpretation of the dendrite growth of Zn electrodeposition during concentration polarization. Inset is the equation of Sand time τ: z is the charge number of the cation, D represents the ambipolar diffusion constant, J is the current density, c is the initial concentration of cations, F is the Faraday's constant, and ta stands for the anion transference number. (For GF and GF@IZO, the same data are: z = 2; D; J; c; F) The anion transference number of GF is 0.63, and the anion transference number of GF@IZO is 0.13. τ(GF): τ(GF@IZO) = 0.043<1, so the GF@IZO increase the Sand' time, consequently mitigating dendritic growth.

$$\tau = \pi D\frac{z^2c^2F^2}{4J^2{t_a}^2}$$

Characterizations. All XRD pattern was measured by Bruker D8 Discover X-ray diffractometer with Cu K α radiation (λ = 1.5418 Å). SEM images were collected by a JEOL-7100F microscope with an acceleration voltage of 20 kV. Raman spectrum measurement was implemented by a Renishaw RM-1000 laser Raman microscopy system. X-ray photoelectron spectroscope (XPS) measurement was achieved by using the VG K-Alpha Probe spectrometer (Thermofisher Scientific) with Al Ka radiation as the excitation source. The electrolyte intrinsic properties were characterized by Fourier transform infrared spectroscopy (FTIR, Nicolt iS50, Thermo Fisher, American). The properties of the composition in Zn anode were evaluated by time of flight-secondary ion mass spectrometer (ToF-SIMs, nano TOFIII, Ulvac-Phi, Japan).

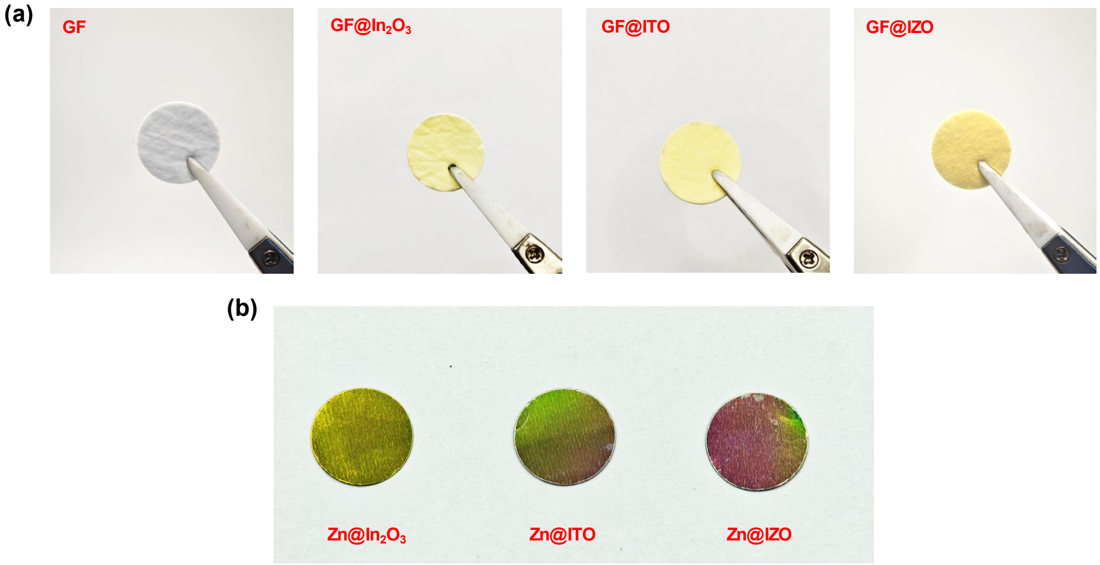

Figure S1. (a) Optical pictures of the GF, GF@In_2O_3, GF@ITO and GF@IZO. (b) Optical pictures of the Zn@In_2O_3, Zn@ITO and Zn@IZO.

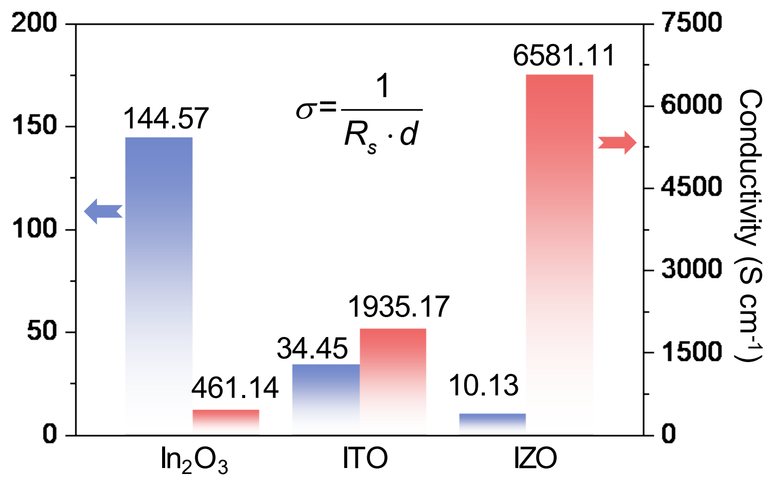

Figure S2. Sheet resistance (R_s) and conductivity (σ) test with In_2O_3, ITO and IZO. Where d represents the thickness of the separator, R represents the resistance according to EIS measurement, and S is the contact area between the separator and electrodes.

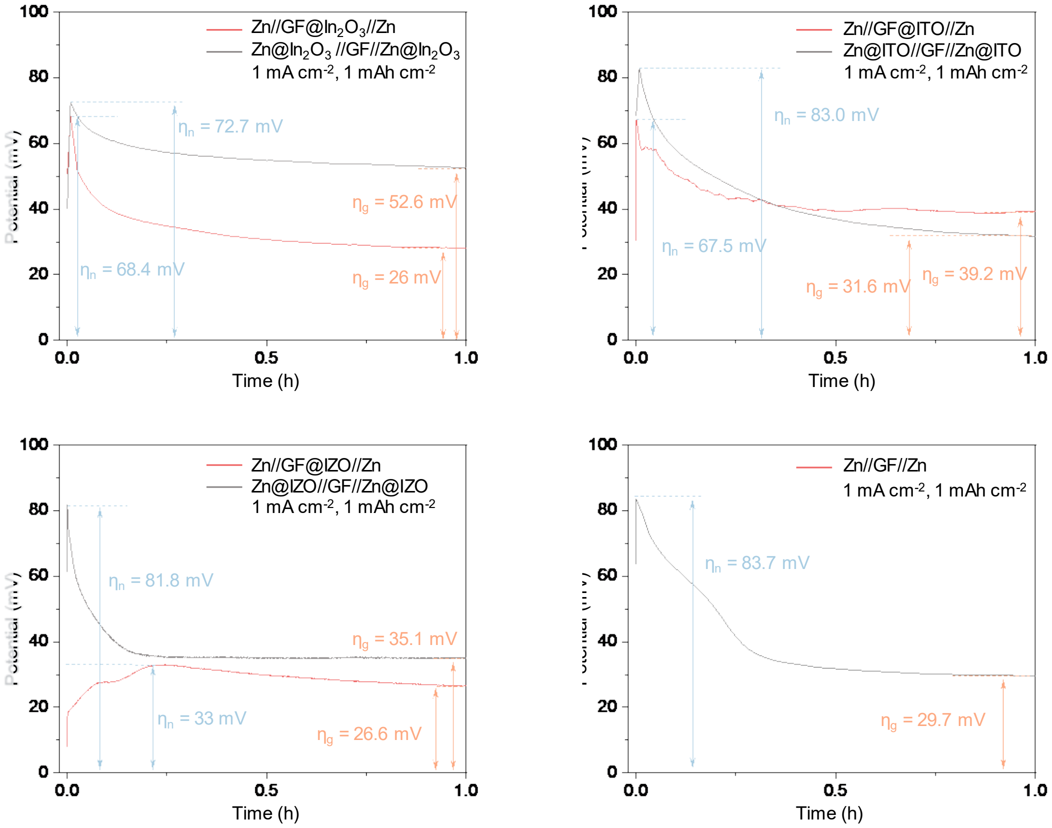

Figure S3. (a-d) Nucleation overpotential and growth overpotential of GF, GF@In_2O_3, GF@ITO, and GF@IZO.

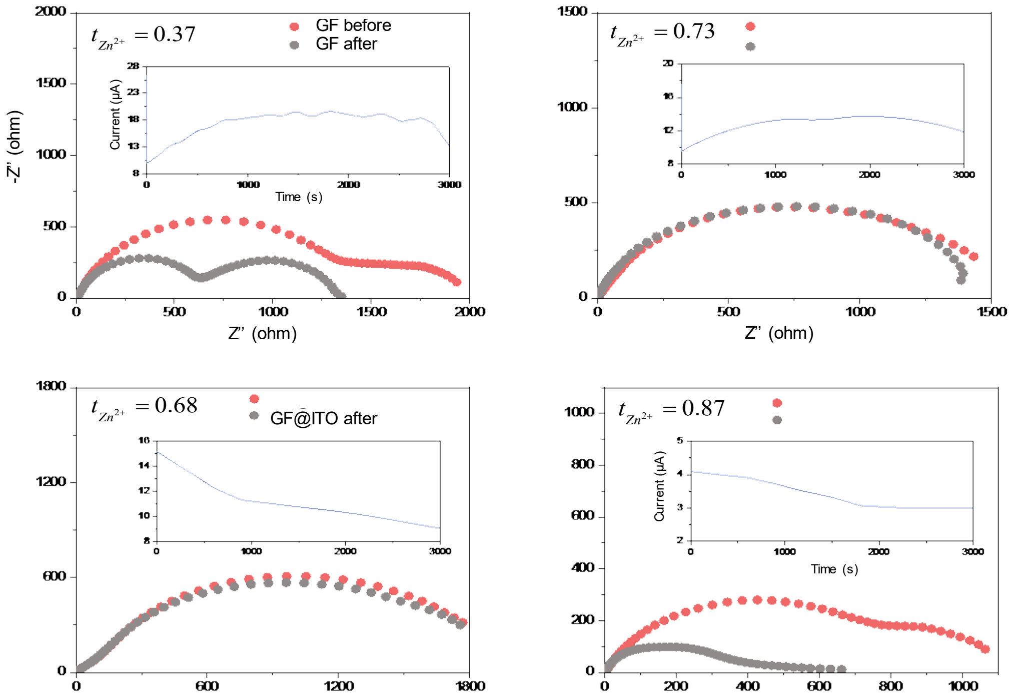

Figure S4. (a-d) EIS of the Zn||Zn symmetrical cells with GF, GF@In_2O_3, GF@ITO, and GF@IZO before polarization and after polarization at a constant potential (10 mV). Corresponding current-time plots in the each EIS.

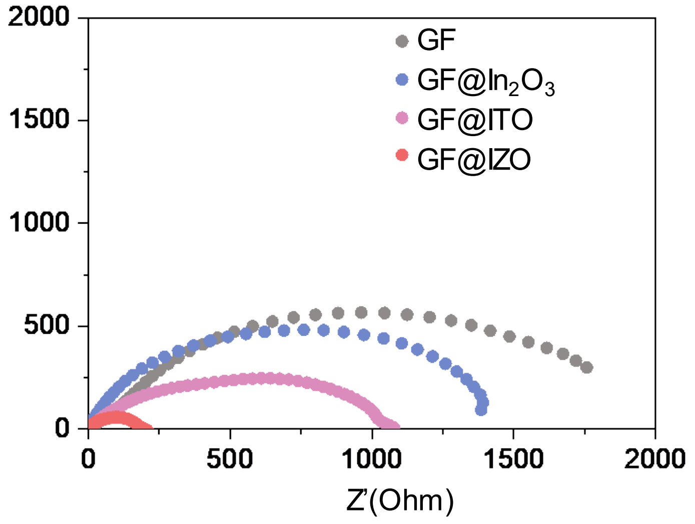

Figure S5. Nyquist EIS plots of Zn-Zn symmetrical cells with GF and GF@IZO.

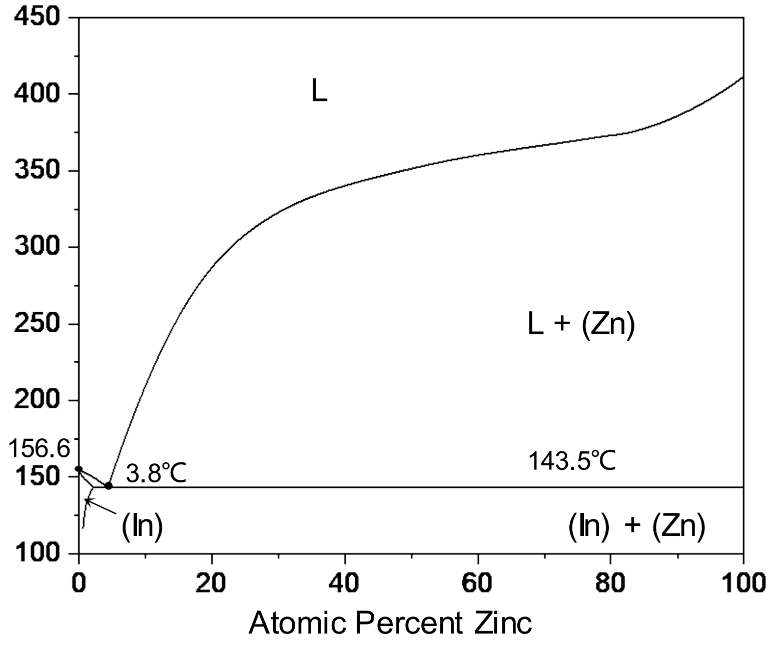

Figure S6. Phase diagrams of Zn with indium[5].

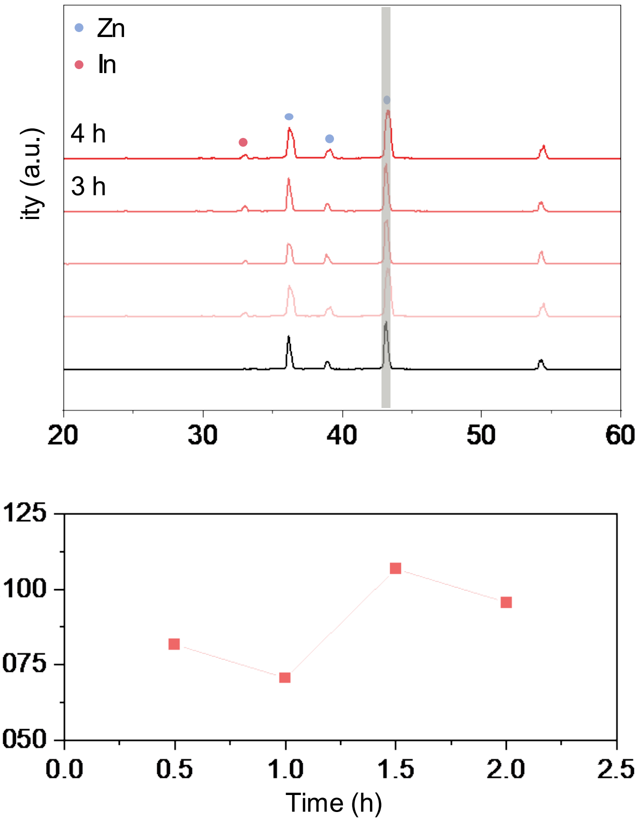

Figure S7. (a) Corresponding XRD spectra. (b) The change in the ratio of In/Zn (101).

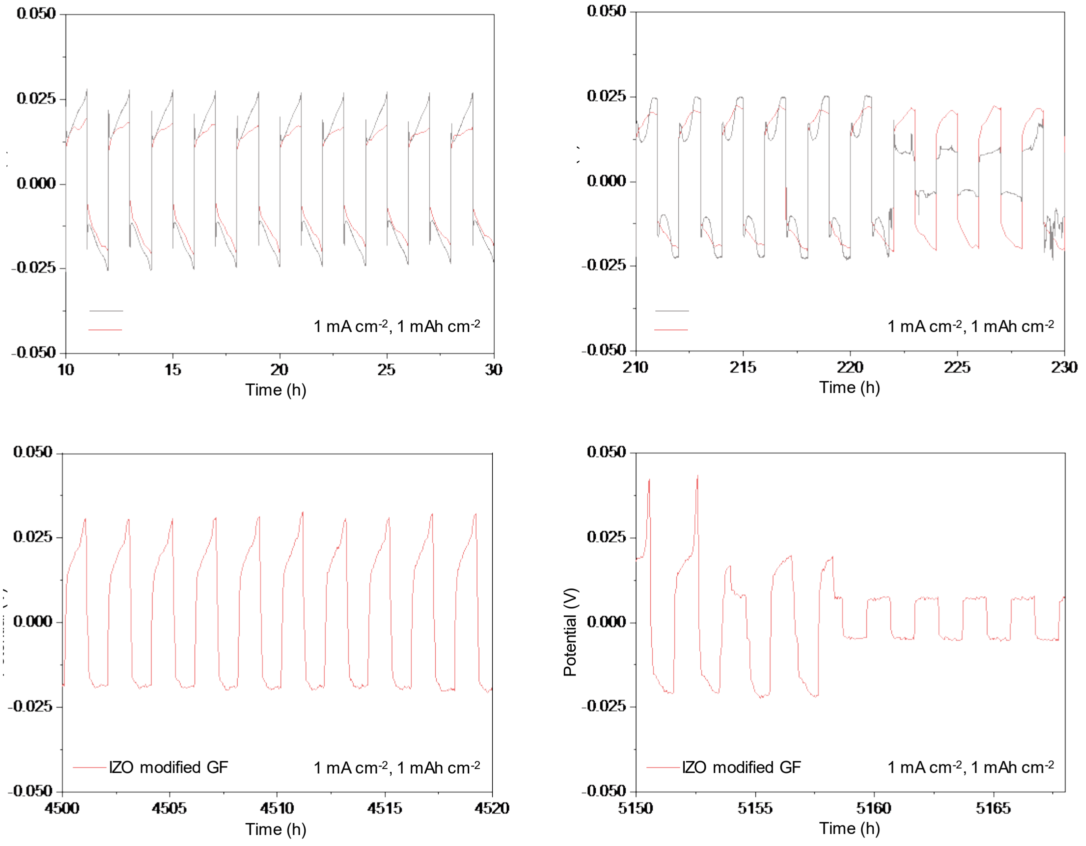

Figure S8. Details of GF and GF@IZO in Zn-Zn symmetrical cells at 1 mA cm^{-2}@1 mAh cm^{-2}.

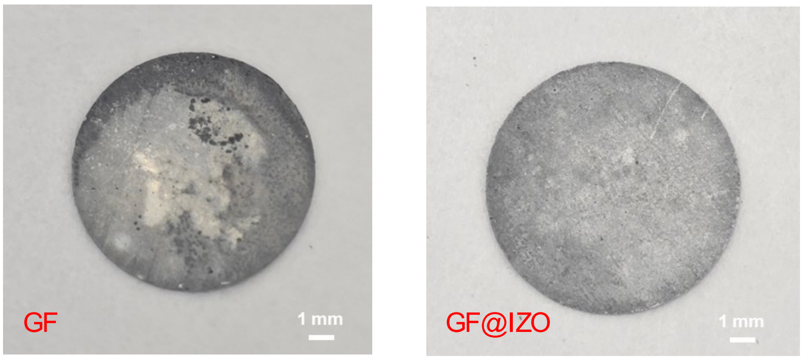

Figure S9. Optical pictures of Zn anode after 200 h plating/stripping assembled with GF separator and GF@IZO separator.

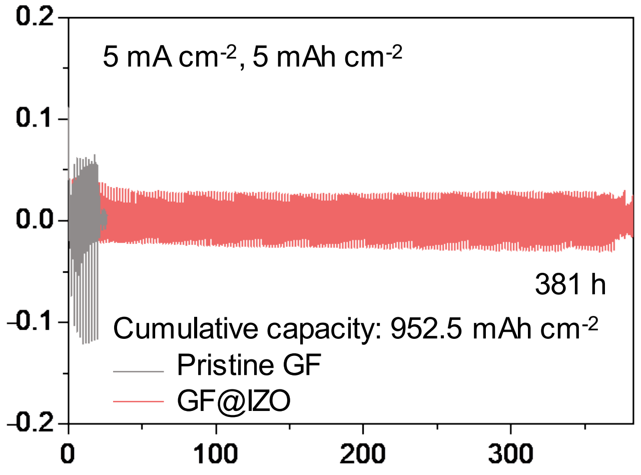

Figure S10. Cycling performance of symmetrical cells at 1 mA cm^{-2}@1 mAh cm^{-2}.

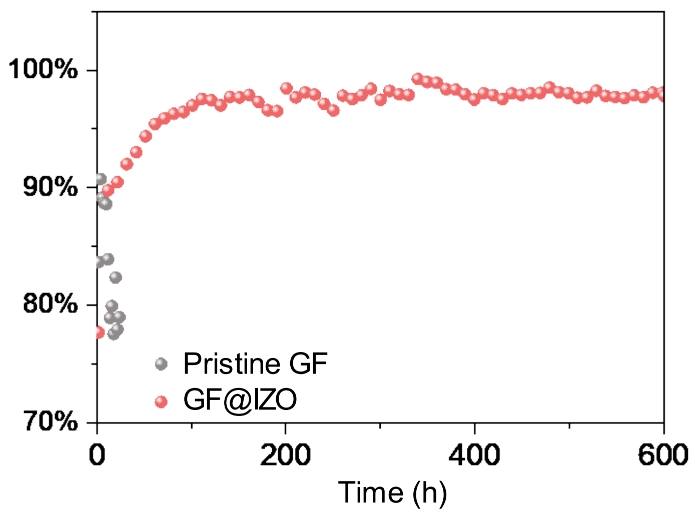

Figure S11. CEs of the Zn-Ti half cells with GF and GF@IZO.

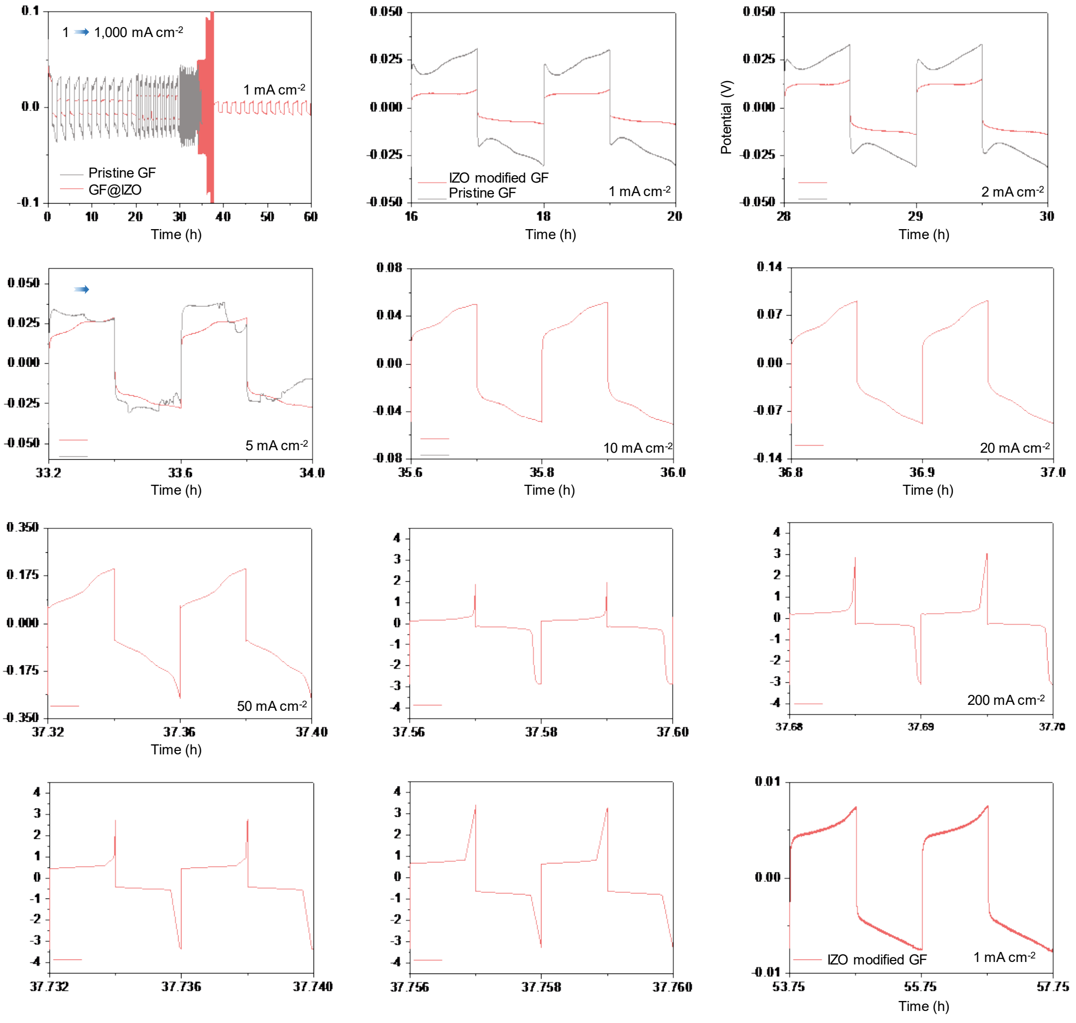

Figure S12. Details of GF and GF@IZO in Zn-Zn symmetrical cells rate performance.

Table S1. Comparison of performance of symmetrical cells involving different separators.

Materials	Lifespan (h)	Transference number	Nucleation overpotential (mV)	Growth overpotential (mV)	CE (%)	Ref
This work	5000	0.87	33	26.6	99	-
SZ	2500	0.64	31.5	29.5	99	[6]
FCNF	2065	0.49	74	30	96.9	[7]
ZSM-5	2000	0.72	27.74	45.74	98.9	[8]
Janus	1400	0.826	39.3	38.5	99.6	[9]

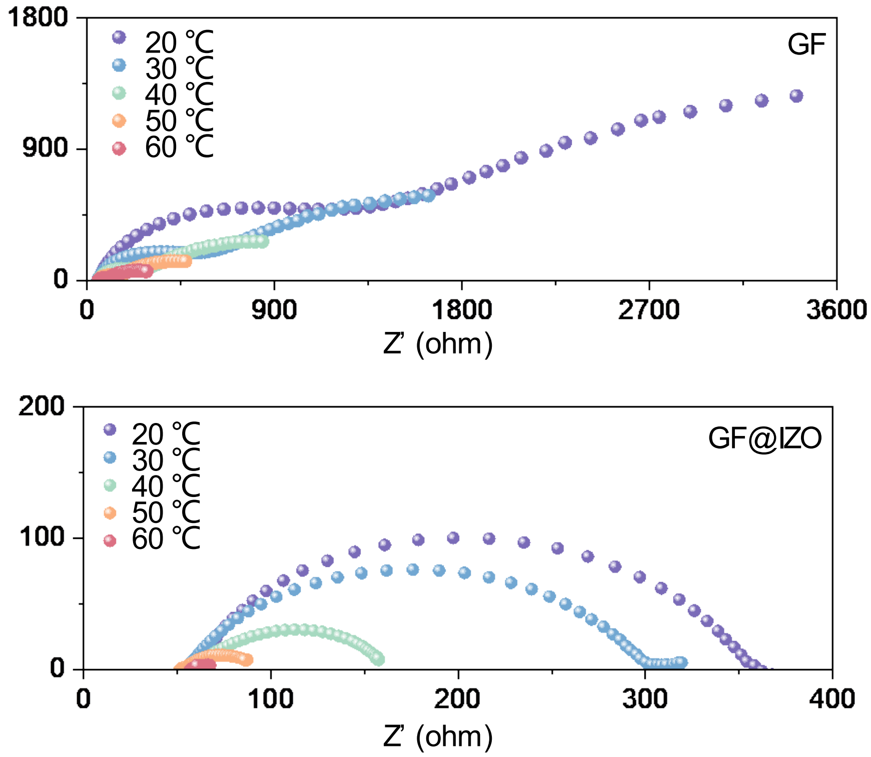

Figure S13. EIS measurement on Zn-GF-Zn and Zn-GF@IZO-Zn symmetrical cells at different temperatures.

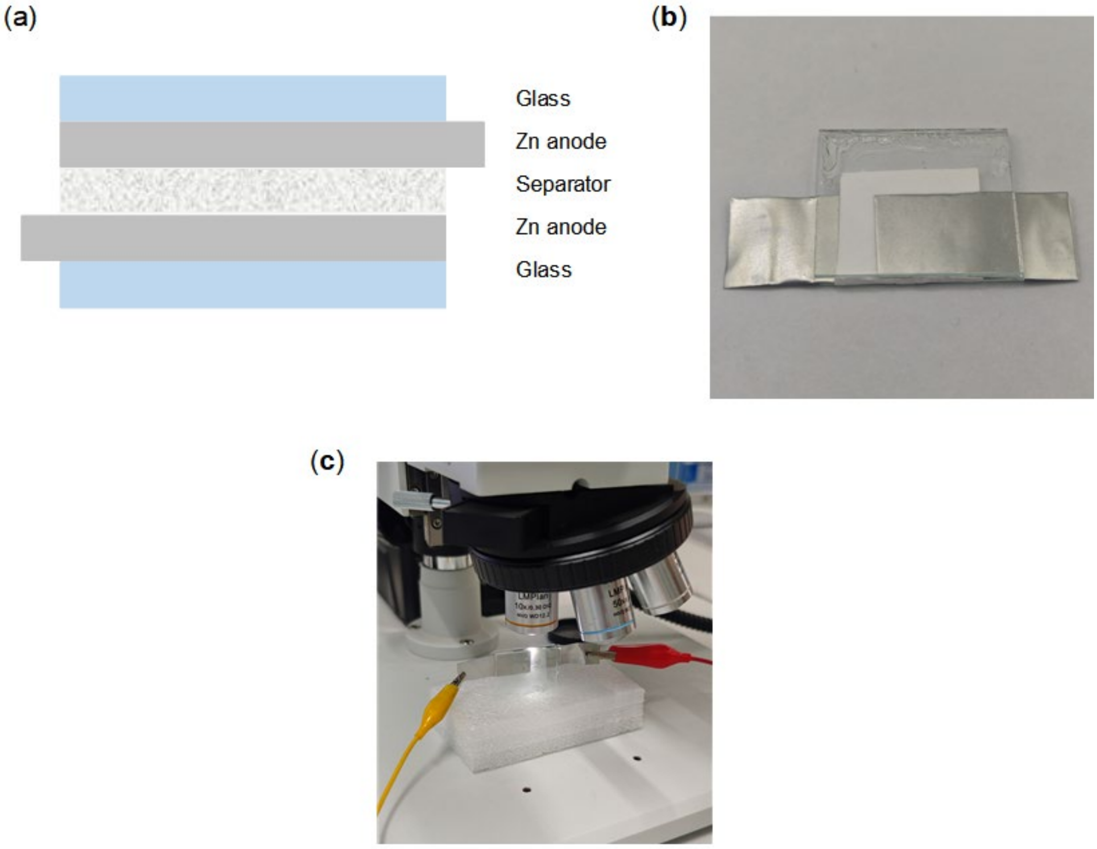

Figure S14. (a) Model of the test device; (b) Optical diagram of the test device; (c) Diagram of in-situ optical microscopy test.

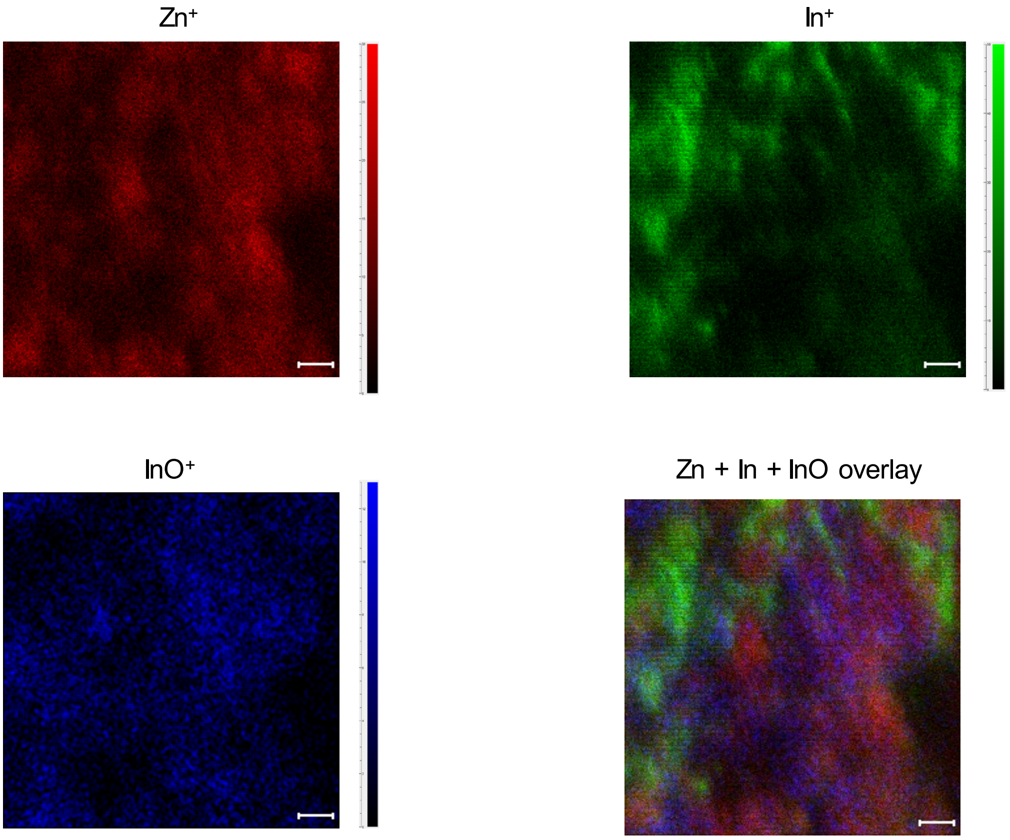

Figure S15. TOF-SIMS pictures of In-Zn hybrid interface.

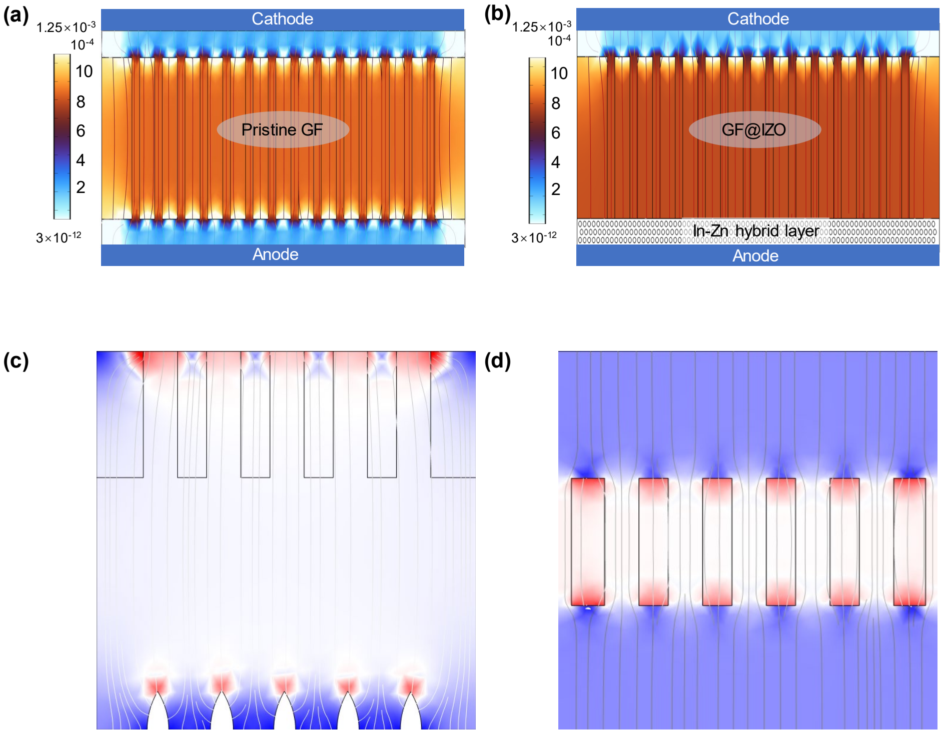

Figure S16. Comsol of electric field distribution.

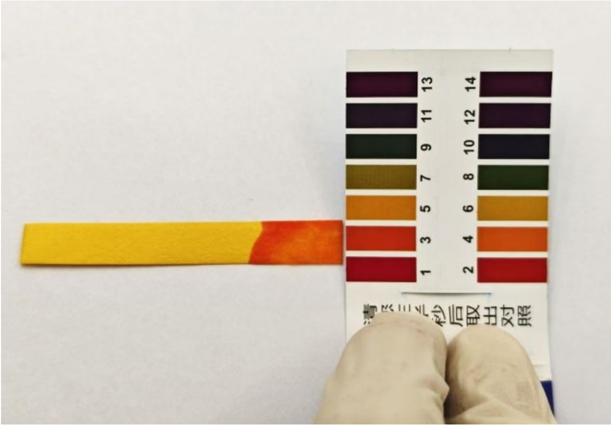

Figure S17. PH value test of GF@IZO after immersion in $Zn(OTf)_2$ for 24 hours.

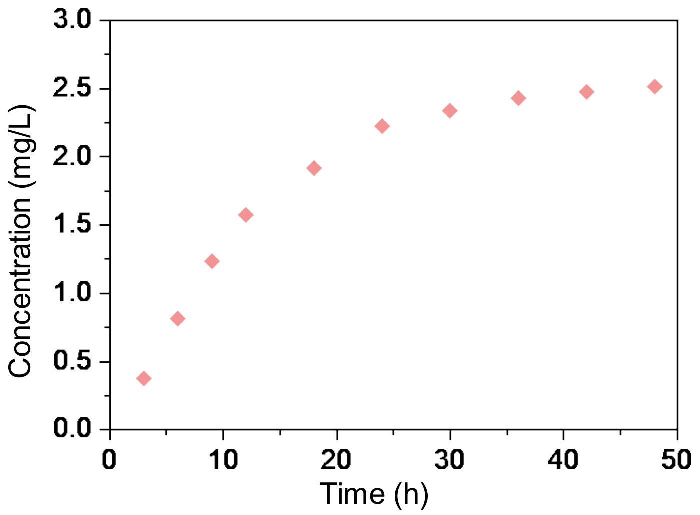

Figure S18. ICP test of pristine GF and GF@IZO after immersion in $Zn(OTf)_2$ for 24 hours.

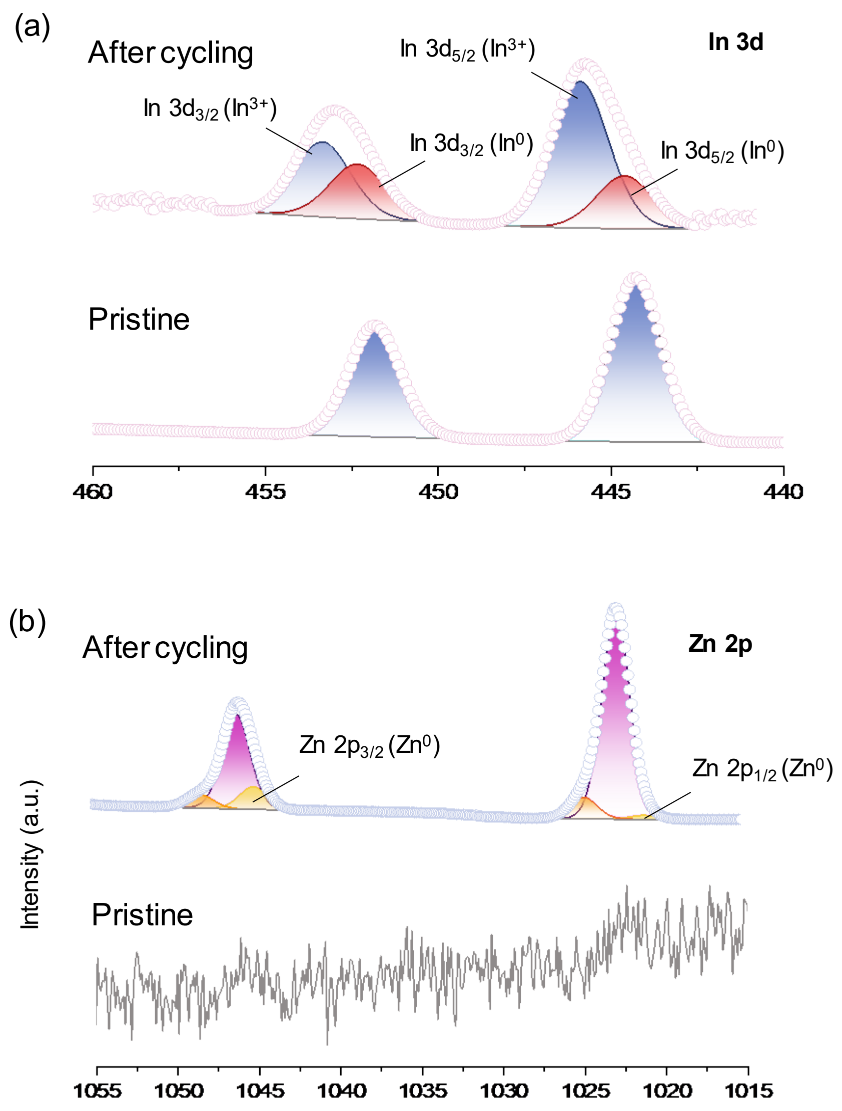

Figure S19. XPS spectrum of the a) In 3d core level and b) Zn 2p core level in surface of GF@In_2O_3 assembled Zn-Zn symmetrical cells before and after cycling.

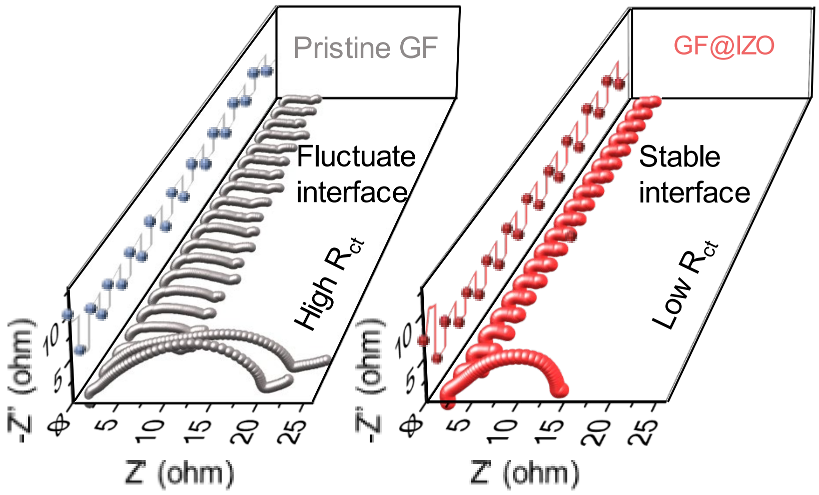

Figure S20. In situ EIS test for GF and GF@IZO.

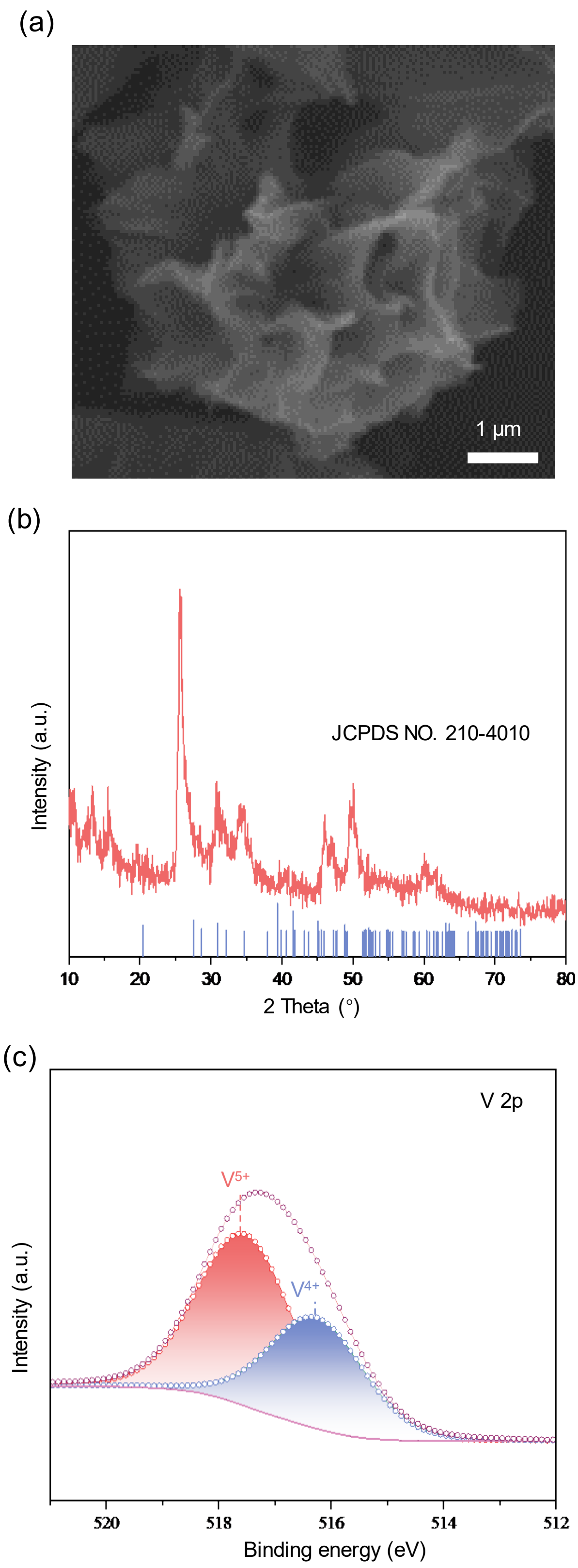

Figure S21. Structure of the synthesized V_6O_{13}. a) SEM image. b) XRD pattern. c) XPS spectrum of the V 2p core level in V_6O_{13}.

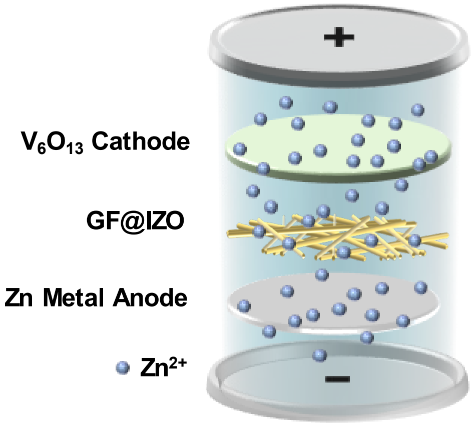

Figure S22. Schematic device structure of battery.

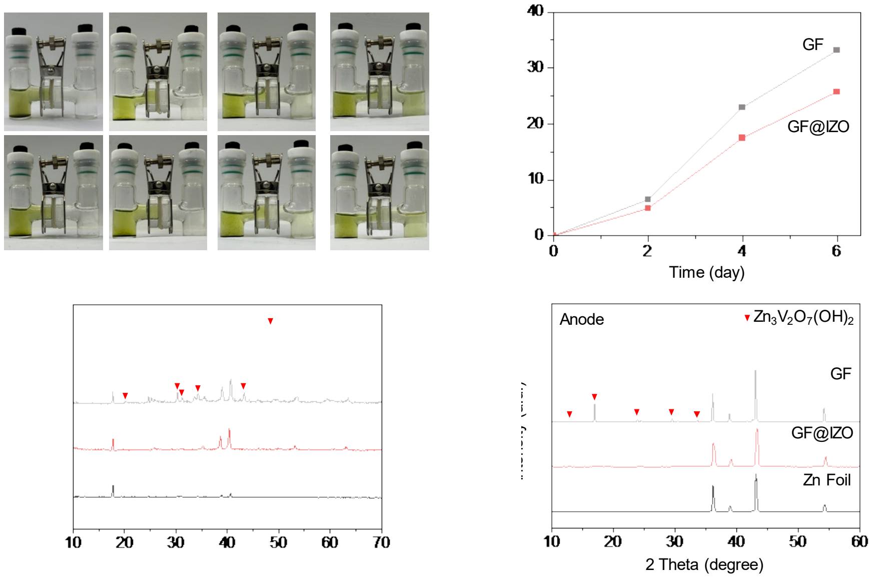

Figure S23. a) Visualized penetration experiment of V ions demonstrated by H-type glass cell: the left chamber contained a yellow-colored V-solution, while the right chamber was filled with colorless water, and separated by GF or GF@IZO. (b) V ions concentration in the right chamber of H-type cells. (c) Ex situ XRD patterns of V-cathode after charging to 1.5 V, and (d) the corresponding XRD pattens of the Zn anode.

H-type glass cells are assembled to visually monitor the penetration of vanadium ions over time (Figure S23a). The concentration of V^{5+} in the right chamber is quantified by ICP-OES. We observe that the color evolution in the right chamber was highly similar for both the pristine GF and GF@IZO. As depicted in Figure S23b, a slightly lower number of vanadium ions cross from the left chamber to the right with the GF@IZO, in contrast to the pristine GF. These results demonstrate that the GF@IZO functions mainly as a physical barrier against vanadium ions. In the full battery system, dissolved vanadium ions react spontaneously with Zn^{2+} in the electrolyte to form zinc vanadate (ZVO) on the electrode surfaces. XRD is employed to identify the presence of ZVO on the electrodes. For reference, the standard diffraction peaks of ZVO (PDF#50-0570) appear at 20.3°, 30.2°, 31.3°, 34.2°, respectively. When equipped with the GF@IZO, only weak diffraction peaks corresponding to ZVO are detected on the cathode surface (Figure S23c), and no characteristic ZVO peaks are observed on the anode surface (Figure S23d). In sharp contrast, distinct ZVO peaks appears on both the cathode and anode when using the GF. These surface-derived by-products induce low Coulombic efficiency and a structurally disordered Zn surface, which aggravate Zn dendrite growth and degrade cycling stability. Overall, these results indicate that the GF@IZO slightly suppresses the diffusion of vanadium ions and slows the formation of ZVO on the cathode. In comparison, its protective effect on the anode is more pronounced.

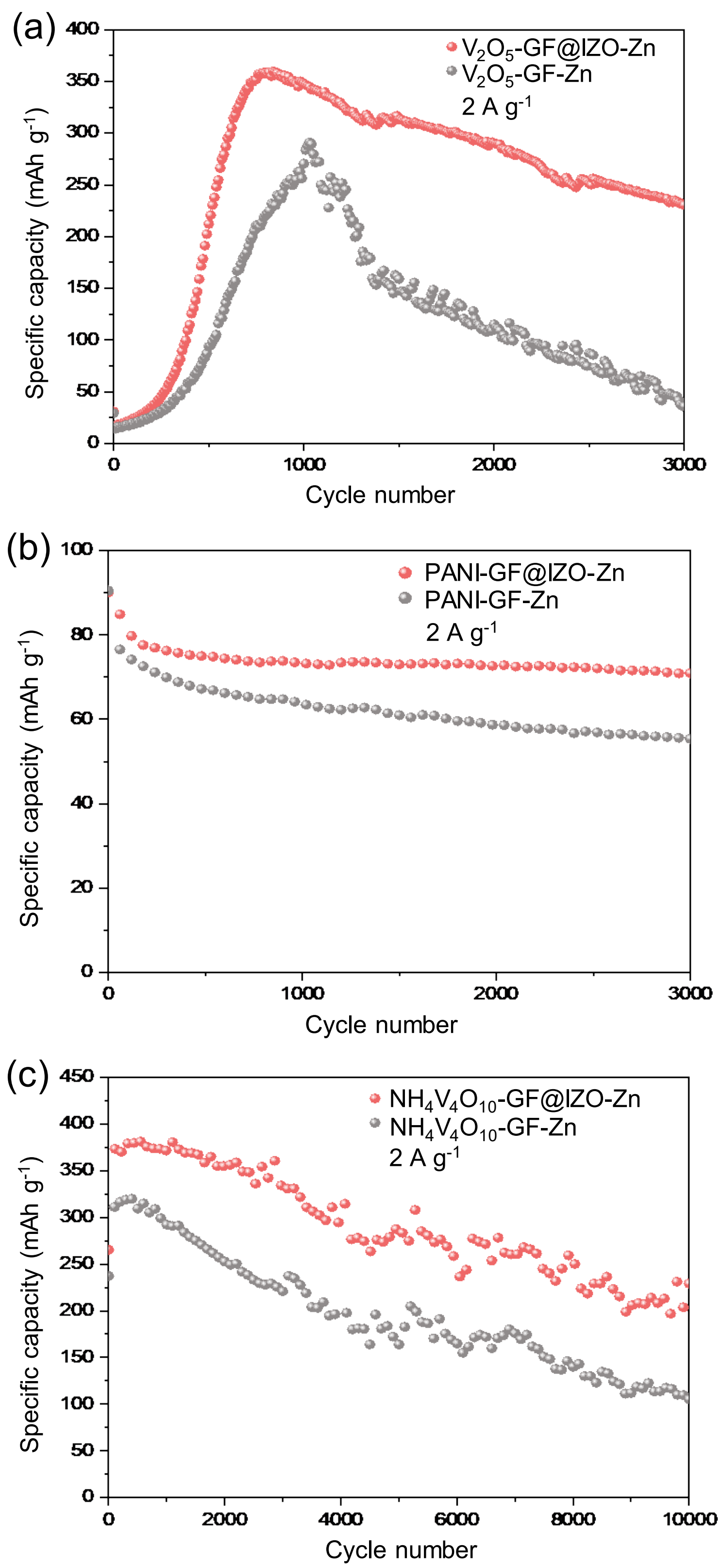

Figure S24. Cycling performance of a) V_2O_5-GF@IZO-Zn and V_2O_5-GF-Zn b) PANI-GF@IZO-Zn and PANI-GF-Zn c), $NH_4V_4O_{10}$-GF@IZO-Zn and $NH_4V_4O_{10}$-GF-Zn at 2 A g^{-1}.

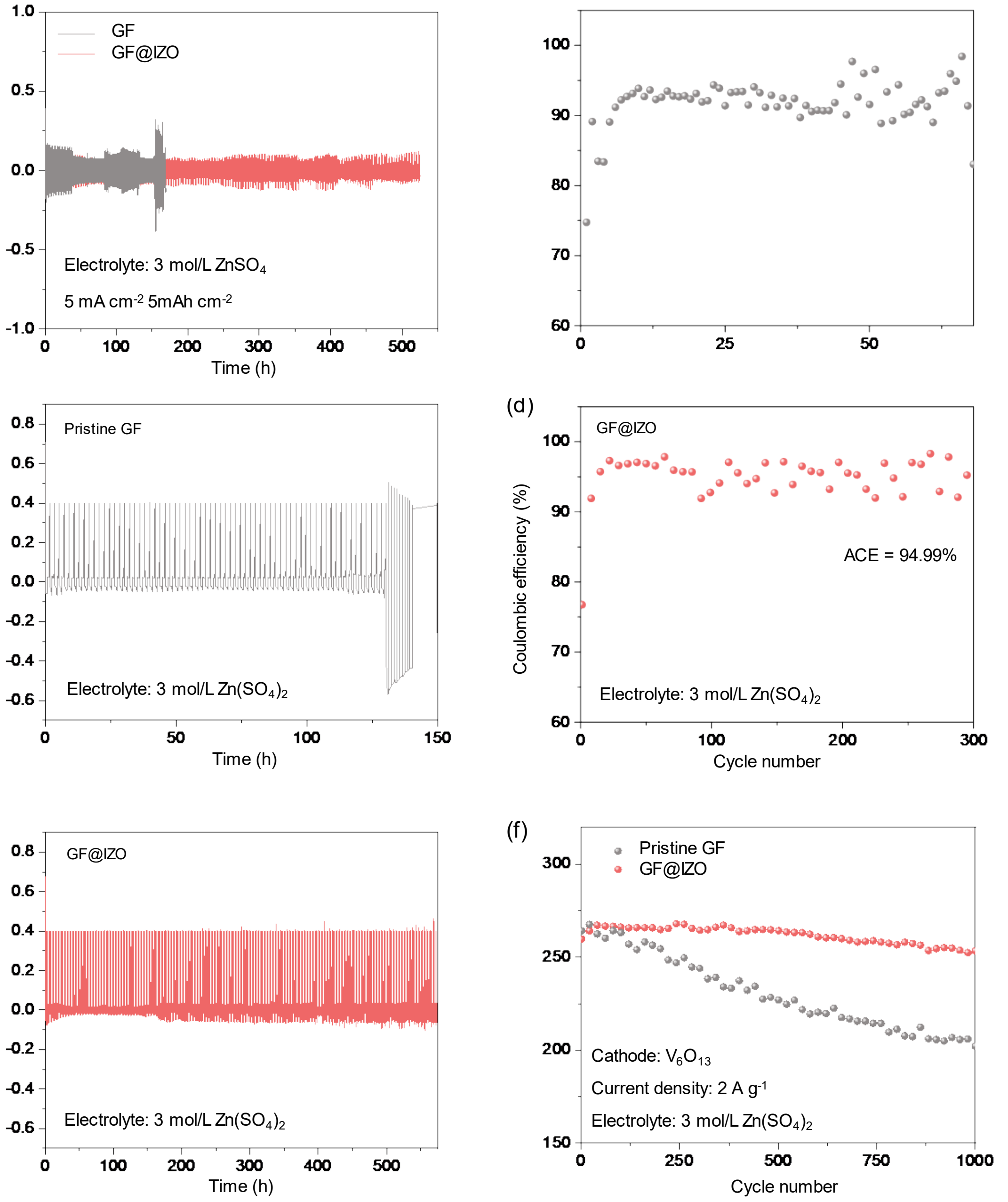

Figure S25. Cycling performance with 3 mol/L $Zn(SO_4)_2$. (a) Symmetrical cells at 5 mA cm^{-2}@5 mAh cm^{-2}. CEs of the Zn-Ti half cells with (b-c) GF and (d-e) GF@IZO. (f) VO_X full cells at 2 A g^{-1}.

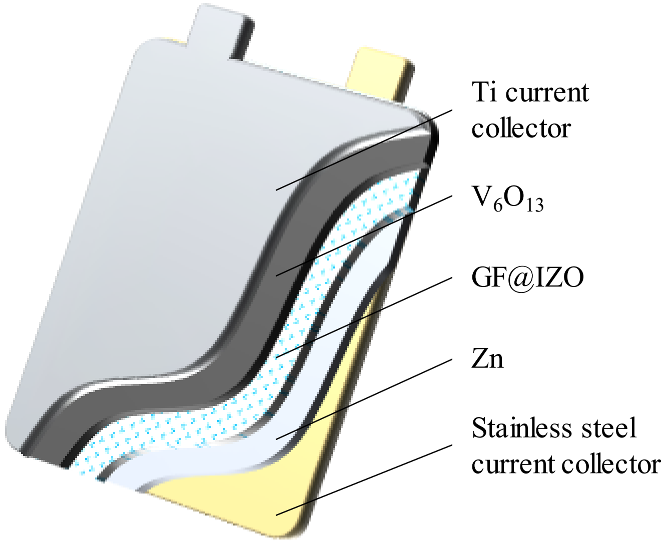

Figure S26. Schematic device structure of pouch cell.

Table S2. Comparison of performance of symmetrical cells involving interfacial modification.

Materials	Electrolyte	Capacity retention/Cycle number/Current density	Ref
This work	3 M $Zn(OTf)_2$	1/3000/2A	-
	3 M $Zn(OTf)_2$	0.726/7110/2A	
A-Zn@CNTs	1 M $ZnSO_4$	0.743/1000/1A	[10]
ZWO@ZN	2M $ZnSO_4$	0.873/1000/1A	[11]
PAZPM	1 M $ZnSO_4$	0.880/1000/5A	[12]
the sulfonic cellulose-grafted/graphene -coated Janus separator	3M $ZnSO_4$	0.9548/1900/1A	[13]
PAM+45% Tre	2M $ZnSO_4$	0.6227/3000/5A	[14]
zeolite-modified	2M $ZnSO_4$	0.703/3000/2A	[15]
MesoTi3C2-wrapped PP separator	2M $ZnSO_4$	0.7625/3000/10A	[16]
ZSM-5	ZSM-5	0.8267/3000/1A	[8]

Reference

[1] Y. Dai, C. Zhang, J. Li, X. Gao, P. Hu, C. Ye, H. He, J. Zhu, W. Zhang, R. Chen, W. Zong, F. Guo, I. P. Parkin, D. J. L. Brett, P. R. Shearing, L. Mai, G. He, *Advanced Materials* **2024**, *36*, 2310645.

[2] X. Wang, Y. Wang, A. Naveed, G. Li, H. Zhang, Y. Zhou, A. Dou, M. Su, Y. Liu, R. Guo, C. C. Li, *Adv. Funct. Mater.* **2023**.

[3] P. Luo, F. Chao, C. Zuo, W. Zhang, F. Xiong, Z. Huang, D. Zhu, G. Yu, W. Zhong, X. Chen, H. Tang, X. Wei, Q. An, *Nano Res.* **2024**, *17*, 6168.

[4] Z. Hou, Y. Gao, H. Tan, B. Zhang, *Nat Commun* **2021**, *12*, 3083.

[5] J. Dutkiewicz, W. Zakulski, *Bulletin of Alloy Phase Diagrams* **1984**, *5*, 284.

[6] X. Wang, Y. Qin, **2023**, DOI 10.21203/rs.3.rs-3062213/v1.

[7] Y. Li, X. Peng, X. Li, H. Duan, S. Xie, L. Dong, F. Kang, *Advanced Materials* **2023**, *35*, 2300019.

[8] J. Zhu, Z. Bie, X. Cai, Z. Jiao, Z. Wang, J. Tao, W. Song, H. J. Fan, *Advanced Materials* **2022**, *34*, 2207209.

[9] X. Zhang, J. Li, K. Qi, Y. Yang, D. Liu, T. Wang, S. Liang, B. Lu, Y. Zhu, J. Zhou, *Advanced Materials* **2022**, *34*, 2205175.

[10] Y. Shao, Z. Xia, L. Xu, X. Zhang, D. Yang, Z. Yang, J. Luo, G. Xiao, Y. Yang, Y. Su, G. Lu, J. Sun, T. Cheng, Y. Shao, *Advanced Materials* **2024**, 2407143.

[11] J. Cao, H. Wu, D. Zhang, D. Luo, L. Zhang, X. Yang, J. Qin, G. He, *Angew Chem Int Ed* **2024**, *63*, e202319661.

[12] K. Zhu, X. Niu, W. Xie, H. Yang, W. Jiang, M. Ma, W. Yang, *Energy Environ. Sci.* **2024**, *17*, 4126.

[13] X. Zhang, J. Li, K. Qi, Y. Yang, D. Liu, T. Wang, S. Liang, B. Lu, Y. Zhu, J. Zhou, *Advanced Materials* **2022**, *34*, 2205175.

[14] S. Yang, Q. Wu, Y. Li, F. Luo, J. Zhang, K. Chen, Y. You, J. Huang, H. Xie, Y. Chen, *Angew Chem Int Ed* **2024**, *63*, e202409160.

[15] H. Yang, Y. Qiao, Z. Chang, H. Deng, X. Zhu, R. Zhu, Z. Xiong, P. He, H. Zhou, *Advanced Materials* **2021**, *33*, 2102415.

[16] F. Bu, Z. Sun, W. Zhou, Y. Zhang, Y. Chen, B. Ma, X. Liu, P. Liang, C. Zhong, R. Zhao, H. Li, L. Wang, T. Zhang, B. Wang, Z. Zhao, J. Zhang, W. Li, Y. S. Ibrahim, Y. Hassan, A. Elzatahry, D. Chao, D. Zhao, *J. Am. Chem. Soc.* **2023**, *145*, 24284.
